# Vaccinia Virus Infection Requires Maturation of Macropinosomes

**DOI:** 10.1111/tra.12290

**Published:** 2015-05-06

**Authors:** Zaira Rizopoulos, Giuseppe Balistreri, Samuel Kilcher, Caroline K. Martin, Mohammedyaseen Syedbasha, Ari Helenius, Jason Mercer

**Affiliations:** ^1^ ETH Zürich Institute of BiochemistryOtto‐Stern‐Weg 38093 ZürichSwitzerland; ^2^ MRC‐Laboratory for Molecular Cell BiologyUniversity College LondonGower StreetLondon WC1E 6BTUK

**Keywords:** endocytosis, macropinocytosis, phosphoinositide exchange, PIKfyve, Poxvirus, Rab conversion, virus entry

## Abstract

The prototypic poxvirus, vaccinia virus (VACV), occurs in two infectious forms, mature virions (MVs) and extracellular virions (EVs). Both enter HeLa cells by inducing macropinocytic uptake. Using confocal microscopy, live‐cell imaging, targeted RNAi screening and perturbants of endosome maturation, we analyzed the properties and maturation pathway of the macropinocytic vacuoles containing VACV MVs in HeLa cells. The vacuoles first acquired markers of early endosomes [Rab5, early endosome antigen 1 and phosphatidylinositol(3)P]. Prior to release of virus cores into the cytoplasm, they contained markers of late endosomes and lysosomes (Rab7a, lysosome‐associated membrane protein 1 and sorting nexin 3). RNAi screening of endocytic cell factors emphasized the importance of late compartments for VACV infection. Follow‐up perturbation analysis showed that infection required Rab7a and PIKfyve, confirming that VACV is a late‐penetrating virus dependent on macropinosome maturation. VACV EV infection was inhibited by depletion of many of the same factors, indicating that both infectious particle forms share the need for late vacuolar conditions for penetration.

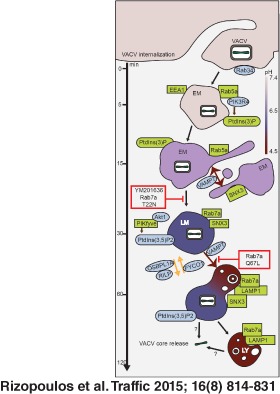

The majority of animal viruses take advantage of cellular endocytic mechanisms to gain entry into their host cells. After internalization, they are ferried into a network of interconnected endocytic vacuoles that provide the necessary cues for the activation of viral fusion/penetration machineries 1, 2. This allows release of viral capsids and genomes into the cytosol. When and where the fusion/penetration events occur depends on the virus, the pathway taken and the nature of ‘cues’ required.

Endocytic vacuoles undergo a gradual maturation that involves changes in composition, cargo content, cytoplasmic location, lumenal milieu, fusion partners, etc. (for recent reviews, see 3 4). The events in the maturation program of endosomes are usually defined and coordinated by Rabs. In the classical endosome pathway, maturation involves Rab5 on early endosomes (EEs) and its exchange for Rab7 on late endosomes (LEs) and lysosomes (LYs) 5, 6. In addition, there is conversion of phosphatidylinositol 3‐phosphate (PtdIns(3)P) to PtdIns(3,5)P_2_, and progressive luminal acidification 7, 8, 9, 10, 11. As virus penetration and uncoating relies on the environment within maturing endosomes, it is perhaps no surprise that for many viruses, infection depends on factors involved in the maturation process 12, 13, 14.

Amongst the endocytic mechanisms used by viruses, macropinocytosis is one of the most common. Macropinocytosis is a triggered process involving complex signaling and vigorous, cell‐wide actin ruffling leading to plasma membrane protrusions. The ruffles can take the form of circular projections, lamellipodia and blebs 15. Macropinosomes are large, fluid‐filled, cytoplasmic vacuoles formed by membrane fission when the protrusions collapse back onto the plasma membrane 16.

Similar to classical endosomes, macropinosomes are known to undergo maturation. The program includes changes in their phosphoinositide (PI) composition, associated Rab GTPases, lumenal pH and localization within the cytoplasm. After detachment from the plasma membrane, the PtdIns(3)P on newly formed macropinosomes is replaced by PtdIns(3,4,5)P_3_ 17. Rab5 accumulates together with its GEF, Rabex5 and effector Rabankyrin 5 17, 18. Macropinosomes may also acquire Rab34 and early endosome antigen 1 (EEA1) needed for pinosome formation and fusion 19, 20. Acquisition of sorting nexins (SNXs) promotes tubulation to facilitate cargo recycling and macropinosome maturation 21, 22, 23, 24, 25. As they move deeper into the cytoplasm, macropinosomes acidify and acquire Rab7, Rab21 and lysosome‐associated membrane protein 1 (LAMP1) 26, 27. While there is no consensus as to the final fate of macropinosomes, it has been shown that they can fuse with each other, LEs and LYs, a process that depends on the PI kinase, PIKfyve 26, 28, 29, 30. Thus, the cargo is eventually degraded.

To date, over 20 different viruses from diverse families have been shown to use macropinocytosis for infectious entry (reviewed in 15 31). Amongst these is vaccinia virus (VACV), a large, enveloped, dsDNA virus characterized by its structural complexity and cytoplasmic life cycle. VACV, the prototypic member of the poxvirus family, was used as the vaccine for the eradication of smallpox 32. During infection, VACV produces two types of infectious particles: mature virions (MVs) with one envelope membrane and extracellular virions (EVs) with two membranes. For internalization and infection, both trigger their own macropinocytosis 33, 34, 35, 36, 37, 38.

The cellular factors required for macropinocytic uptake of VACV MVs and EVs include epidermal growth factor receptor (EGFR) and receptor tyrosine kinase (RTK) and RTK signaling, actin and myosin dynamics, small Rho GTPases and protein kinase C (PKC) as well as PI(3)kinase activity 33, 34, 35, 36, 38, 39. However, the macropinocytic trafficking requirements for VACV infection remain undefined. To this end, we have investigated the pathway taken by VACV MVs and EVs after macropinocytosis in HeLa cells. The maturation of VACV‐induced macropinosomes was analyzed using microscopy in fixed and live cells. A targeted siRNA screen was used to identify cellular factors involved in VACV endocytic trafficking and a variety of perturbations allowed analysis of the critical cellular processes involved.

## Results

### VACV MV‐containing macropinosomes undergo maturation

To investigate the association of VACV with early and late endocytic vacuoles, we allowed MV particles to bind to HeLa cells in the cold and used confocal microscopy to follow the distribution of the virus after warming to 37°C for 0–4 h. The MVs used in these experiments contained a mCherry‐tagged version of the core protein A4 [Western Reserve (WR) mCherry‐A4] 40, which allowed us to visualize individual particles. To distinguish between internalized and surface‐bound virions, we stained those accessible on the plasma membrane of non‐permeabilized cells with the monoclonal antibody (MAb) 7D11, which recognizes the viral membrane protein L1. The HeLa cells expressed EGFP‐tagged versions of EE or LE/LY proteins, Rab5, Rab7 or LAMP1. To localize an additional EE marker, EEA1, cells were permeabilized in some of the experiments and subjected to immunofluorescence staining directed against this factor.

**Figure 1:**
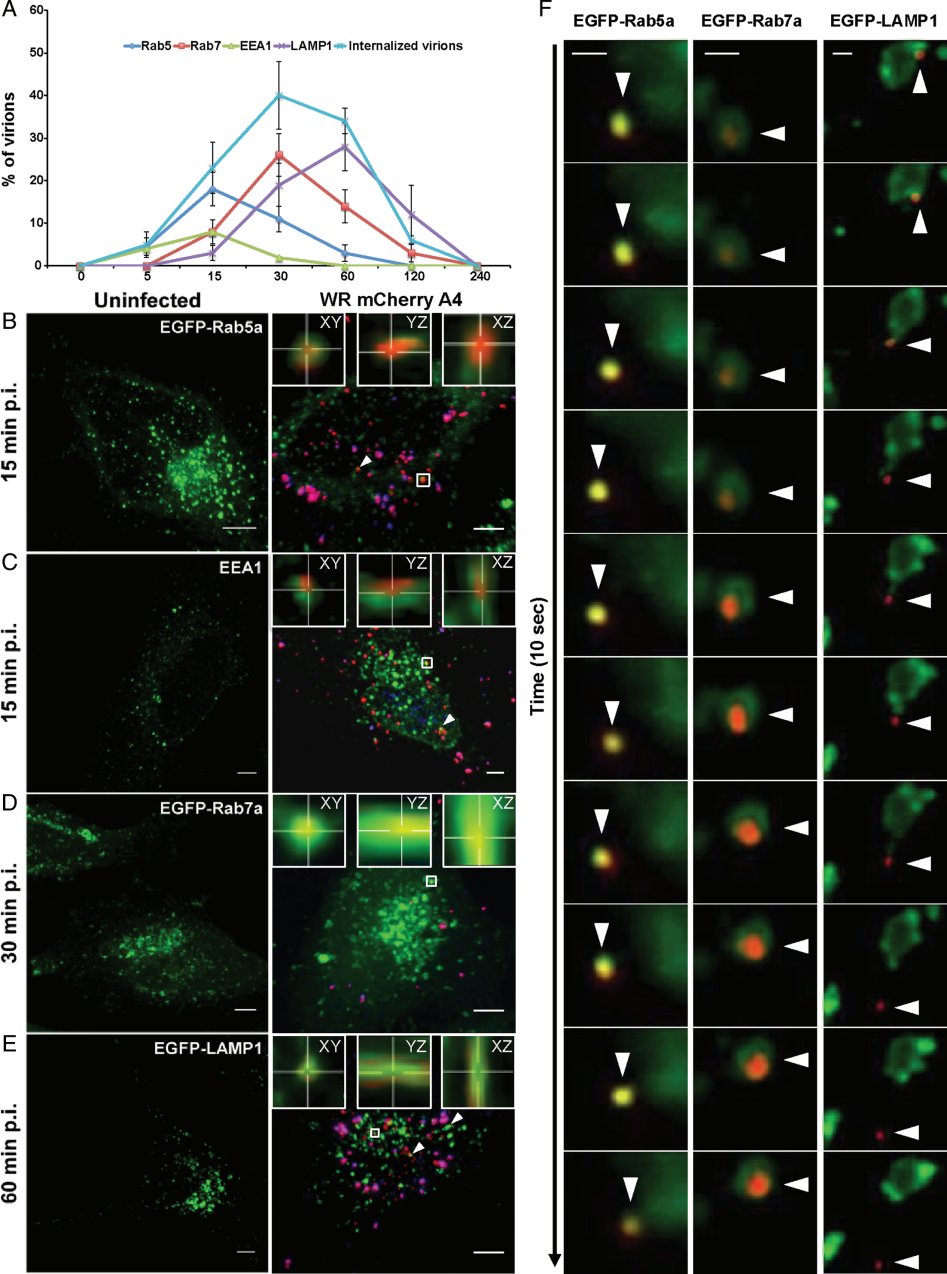
**VACV MVs** **traffic through early and late macropinosomes.** A) The kinetics of internalized MV colocalization with Rab5a, EEA1, Rab7a and LAMP1 were quantified and displayed as percent colocalization at each time point. The light blue line indicates the mean percentage of total virions internalized per cell at each time point. At least 45 cells were analyzed for each marker at all time points. Error bars represent the SD of three independent experiments. B–E) HeLa cells transfected with EGFP‐tagged versions of Rab5a (B), Rab7a (D) or LAMP1 (E) for 18 h. Cells were left uninfected (left panels) or were bound with WR mCherry‐A4 MVs at an MOI of 2 at 4°C (right panels). Cells were washed and shifted to 37°C for various time points (5–240 min) prior to fixation. Non‐permeabilized cells were then subjected to immunostaining with α‐L1R to distinguish bound virions (blue). To visualize endogenous EEA1 (C), cells were permeabilized and immunostained using α‐EEA1 antibody. White arrows represent colocalization events and representative images of the peak time points of colocalization are displayed. Insets display individual co‐localization examples from boxed regions in xy, yz and xz planes (imaris). Scale bars, 5 µm. F) Kymographs of VACV MV movement within Rab5a‐, Rab7a‐ and LAMP1‐positive macropinosomes. Images are derived from 10 second clips of Movies S1 (Rab5a), S3 (Rab7a) and S5 (LAMP1). Scale bars, 2 µm.

When the images were subjected to colocalization analyses using automated particle detection (Figure S1), 40.0% of virions were found to internalize by 30 min (Figure 1A, light blue line), whereafter the number of intracellular virions identifiable as spots began to decline (Figure 1A, light blue line). Penetration and disassembly of the viral cores in the cytosol are known to begin between 20 and 30 min after endocytosis 39, 41. Thus, the subsequent decline in detectable viral spots observed between 60 and 120 min was consistent with the known kinetics of uncoating of the MV genome and core degradation 39, 41, 42.

Incoming viruses were found in Rab5a‐positive, and to a lesser extent in EEA1‐positive, vacuoles within 5 min after warming, indicating that the first maturation step occurred quickly after macropinocytosis (Figure 1A, dark blue and green lines). Colocalization of internalized viruses with these early endocytic markers peaked at 18.0% for Rab5a and 8.0% for EEA1 by 15 min (Figure 1B,C), after which it started to decrease (Figure 1A, Rab5: dark blue line, and EEA1: green line). These results suggested that attachment of these proteins to macropinosomes containing the virus occurred for only a short period after vacuole formation. We noted that the colocalization of virus with EEA1 was lower than Rab5a. This could be due to the fact that EEA1 is only found on a subset of early macropinosomes, as is the case with classical EEs 43. As EEA1 is required for docking and homotypic fusion of early macropinosomes 20, another possibility is that fusion of virus‐containing vacuoles is a rapid event and thus the association of EEA1 with VACV‐containing macropinosomes is brief.

Coincident with the decrease in Rab5a, MV‐containing macropinosomes acquired late macropinosome markers Rab7a and LAMP1. While virions were not detected in Rab7a‐positive macropinosomes at 5 min, colocalization with Rab7a increased to 8.0% by 15 min p.i., peaking at 30 min with 26.0% of MV‐containing macropinosomes (Figure 1A, red line, and 1D). The appearance of LAMP1 was delayed relative to Rab7a, with only 19.0% of virion‐containing vacuoles positive for LAMP1 at 30 min (Figure 1A, purple line). By 60 min, 28.0% of the MV‐positive macropinosomes had acquired LAMP1 (Figure 1E), which then declined over the next 2 h in parallel with the loss of Rab7 (Figure 1A, purple line). In confirmation of these results, a similar percentage of VACV MVs were found to colocalize with endogenous Rab5a (16.7%), Rab7a (26.4%) and LAMP1 (30.6%) at the peak time points of colocalization determined in Figure 1A (15, 30 and 60 min, respectively) (Figure S2).

When HeLa cells transiently expressing EGFP‐tagged Rab5a, Rab7a or LAMP1 were infected with WR mCherry‐A4 MVs and imaged live, coordinated movement of virions within vacuoles positive for these markers was observed (Figure 1F and Movies S1–S6). Live‐cell imaging revealed that infected cells often contained exceptionally large Rab5a‐positive compartments that acquired virus particles through fusion with smaller vesicles (Movies S1 and S2). At later time points, VACV was found within EGFP‐Rab7a and LAMP1‐EGFP vesicles (Movies S3–S6), both of which were capable of homotypic fusion events (Movies S4 and S6). In agreement with the low pH requirement of VACV penetration by fusion out of vacuoles 33, 44, 45, 46, 47, we occasionally observed virus cores escaping from LAMP1‐positive late macropinosomes (Figure 1F and Movie S5).

The results indicated, in accordance with previous reports 39, 46, that VACV MV endocytosis in HeLa cells is rapid and relatively efficient. Vacuoles that contained the internalized virus recruited Rab5a and, in some cases, EEA1 immediately after formation. In the time span of 30–60 min after warming, vacuoles underwent Rab5a to Rab7a conversion followed by the acquisition of LAMP1, an integral membrane protein. Following the acquisition of LAMP1, the total number of internalized cores started to decrease. Consistent with this, when release of cores into the cytosol was observed, it occurred from LAMP1‐containing vacuoles. We have previously shown that virus‐containing vacuoles positive for the endosomal content marker dextran do not contain transferrin at any time 34, 35 confirming that they are distinct from classical endosomes.

### Endocytosis‐directed RNAi screen suggests that macropinosome maturation is critical for VACV infection

To establish which cellular trafficking factors VACV MVs require for infection, we performed a high‐throughput RNAi screen in human tissue culture cells (HeLa) as outlined in Figure S3A. Using a recombinant VACV that expresses EGFP under an early viral promoter (WR E EGFP), we could readily distinguish infected from non‐infected cells based on EGFP expression 6 h post‐infection (h p.i.). Automated fluorescence microscopy and image analysis were employed to score for cell factors required for early stages of infection up to and including translation of early viral genes. The siRNA library contained three siRNAs against 162 human genes known to be involved in endocytosis and endocytic membrane traffic (Table S1) 48.

**Figure 2:**
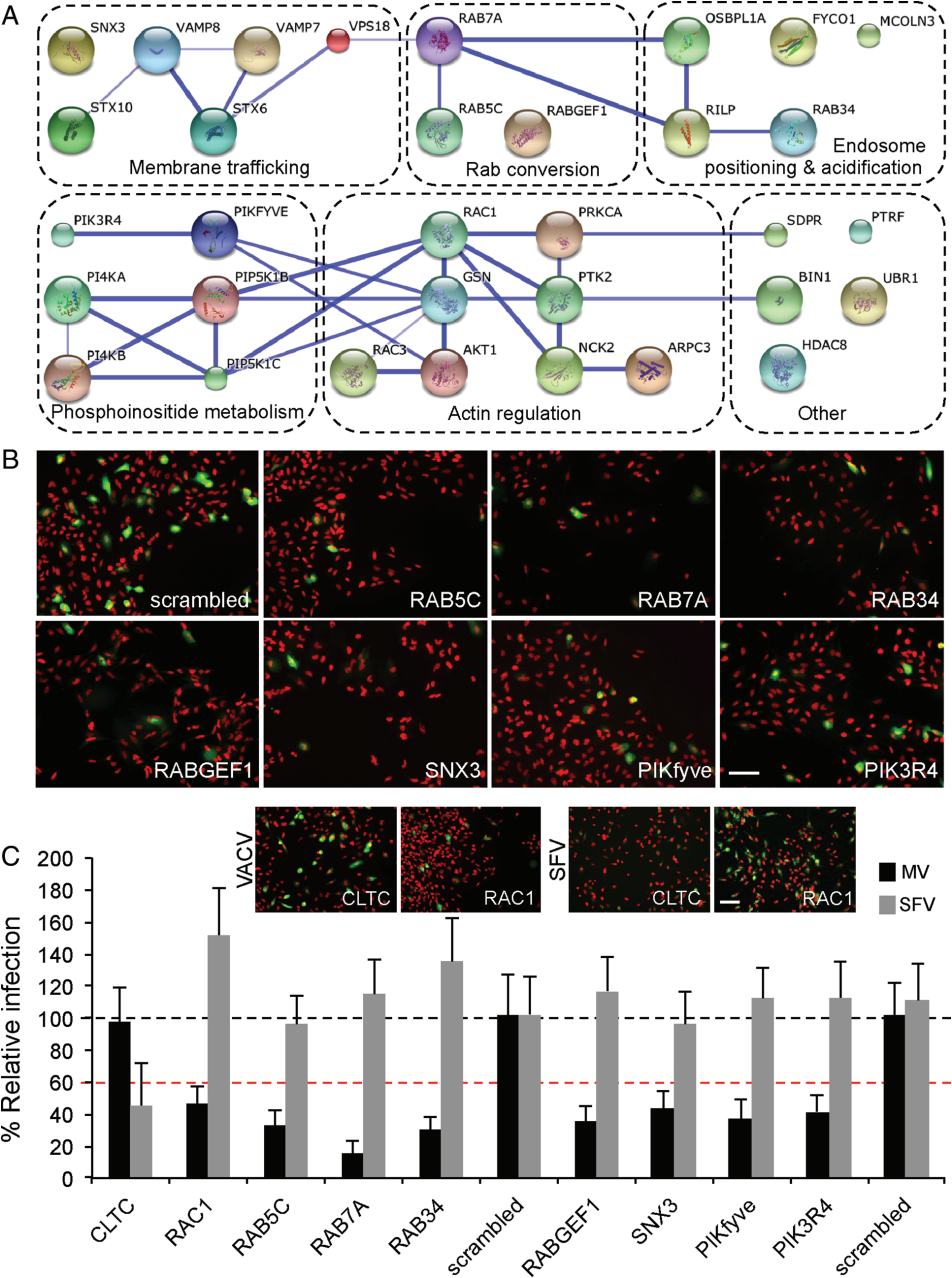
**RNAi** **screen identifies endocytic factors required for** **MV** **macropinocytic trafficking.** A) Host factors required for VACV MV infection are depicted in functional clusters identified by david (dashed boxes). Blue lines between genes indicate high‐confidence (>0.9) string interactions. B) Representative images of a subset of hits from the RNAi screen, nuclei (red), infected cells (green). Bar, 50 µm. C) Comparison of the effects of a subset of VACV MV hits on SFV infection. Depletion of clathrin light chain (CLTC) and Rac1 served as positive controls for SFV and VACV MVs, respectively (comparative images displayed in inset. Scale bar, 50 µm). Results are means of three independent experiments ± SD. Black dashed line is set at 100% relative to scrambled controls. Red dashed line is set at 40%, the cutoff for definition as a hit in the screen.

The screen was performed in triplicate and hits defined as proteins that, when depleted, caused at least a 40% decrease in infection with two or three out of the three siRNAs. Thirty‐three cell factors fulfilled the criteria (Figure 2A and Table S2). When the hits were clustered using our network visualization algorithm 42, which takes in account physical interactions defined in string 49 and cellular functions annotated in david 50, 51, we found that the hits fell into six clusters: membrane trafficking, Rab conversion, endosomal positioning and acidification, PI metabolism and the regulation of actin (Figure 2A and Table S2).

Depletion of Rab5c, Rab7a, Rab34, RABGEF1 (Rabex5), SNX3, PIKfyve and PIK3R4 caused significant inhibition in VACV early gene expression (Figure 2B). To follow up these factors, the siRNA with the greatest effect was rescreened against VACV MVs in parallel with Semliki Forest virus (SFV), an alphavirus that enters cells by clathrin‐mediated endocytosis and penetrates from EEs (Figure 2C). Depletion of clathrin light chain (CLTC), required for SFV, and the small GTPase Rac1 which is required for VACV infection served as controls (Figure 2C; inset). While VACV infection was inhibited by at least 50% in all cases, knockdown of the genes had no effect on SFV infection. That the cells could still support SFV infection indicated that the inhibition of VACV was not due to a general defect in the endosome system or cell fitness. Collectively, the list of validated factors indicated that for infection by VACV MVs, macropinosomes must undergo both early and late stages of maturation.

### VACV infection requires Rab7a function

**Figure 3:**
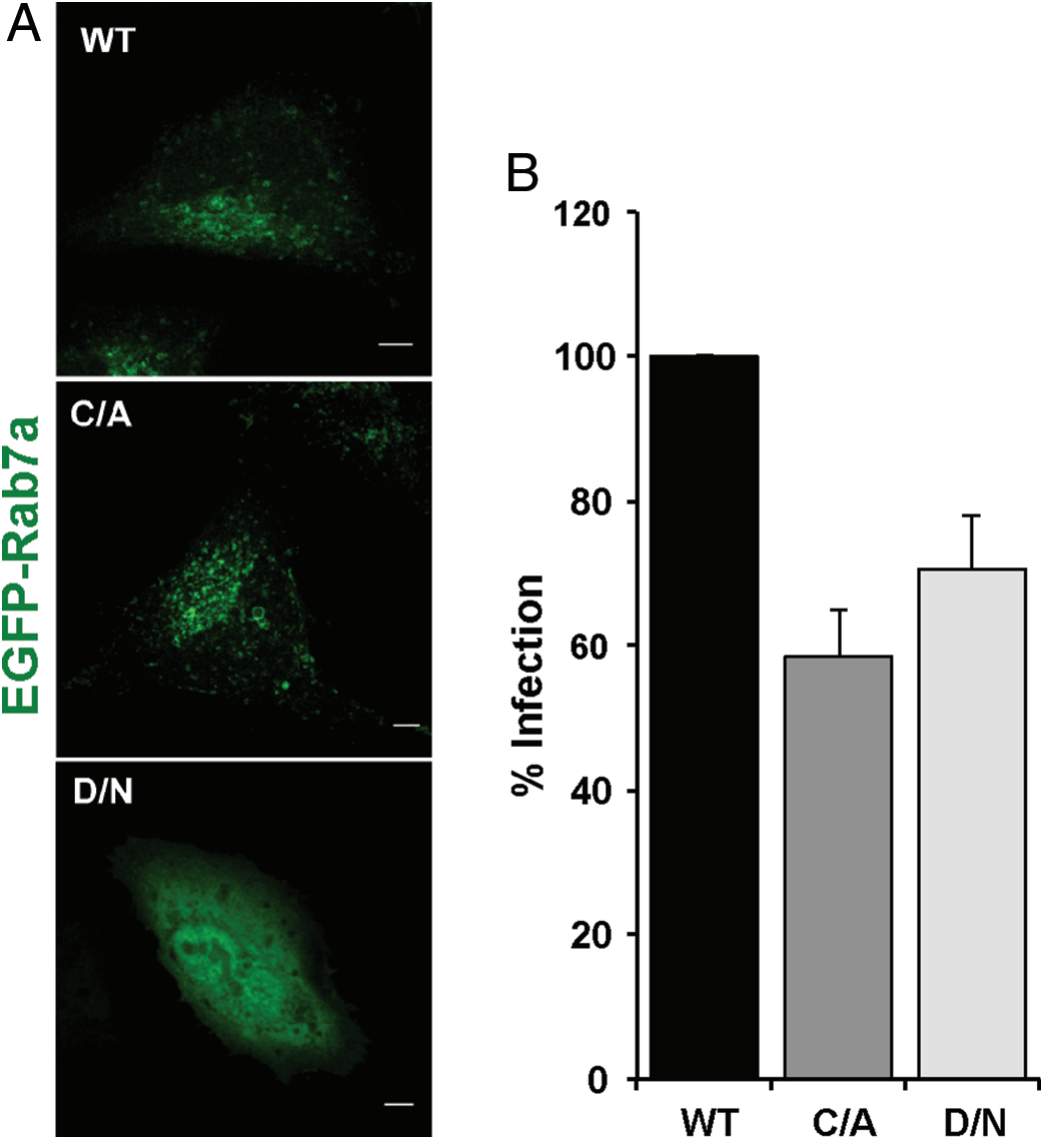
**Rab7a is required for** **VACV MV** **infection.** A) HeLa cells were transfected with WT, C/A or D/N versions of EGFP‐Rab7a. After 18 h, cells were imaged by confocal microscopy. B) HeLa cells transfected with EGFP‐tagged versions of WT, C/A or D/N Rab7a were infected with WR E/L mRFP MVs (MOI = 1). Cells were harvested for flow cytometry 6 h p.i., and 10 000 transfected cells scored for infection. Results are displayed as the percent infection relative to that of WT Rab7a overexpressing cells, and represent the mean of five independent experiments ± SD.

As a classical LE marker, Rab7a is important for LE maturation and lysosome fusion. To determine if Rab7a function was required during VACV trafficking and infection, cells were transfected with wild type (WT), constitutively active (C/A), or dominant negative (D/N) versions of EGFP‐Rab7a. Confocal microscopy of the transfected cell populations showed that the EGFP‐Rab7a variants localized as expected, with WT and C/A Rab7a residing on a perinuclear vesicle population, and D/N Rab7a displaying a diffuse staining throughout the cell (Figure 3A).

The cells were infected with a VACV recombinant expressing mRFP from an early/late promoter (WR E/L mRFP). At 6 h p.i., cells were collected and analyzed by flow cytometry for transfected cells that were also infected. Cells expressing C/A or D/N Rab7a showed a statistically significant (p < 0.05) average decrease in infection of 41.56 and 30.42%, respectively, when compared to cells expressing the WT protein (Figure 3B).

Three Rab7a effectors involved in endosome maturation, Rab‐interacting lysosomal protein (RILP), FYVE coiled‐coil domain‐containing protein 1 (FYCO1), and oxysterol‐binding protein‐like 1A (OSBPL1A), were hits in the RNAi screen directed against VACV MVs (Figure 2). Depletion of the same factors had no inhibitory effect on SFV (not shown). Another hit, SNX3, has been reported to promote recruitment of Rab7a to *Salmonella*‐containing vacuoles (SCVs) 25. Microscopy‐based localization of EGFP‐SNX3 during VACV infection indicated that SNX3 was recruited to MV‐containing vacuoles with similar kinetics as Rab7a (Figure S4). Collectively, the localization, RNAi screening and infection data indicate that recruitment of functional Rab7a to MV‐containing macropinosomes is critical for VACV infection.

### VACV entry depends on PIKfyve

**Figure 4:**
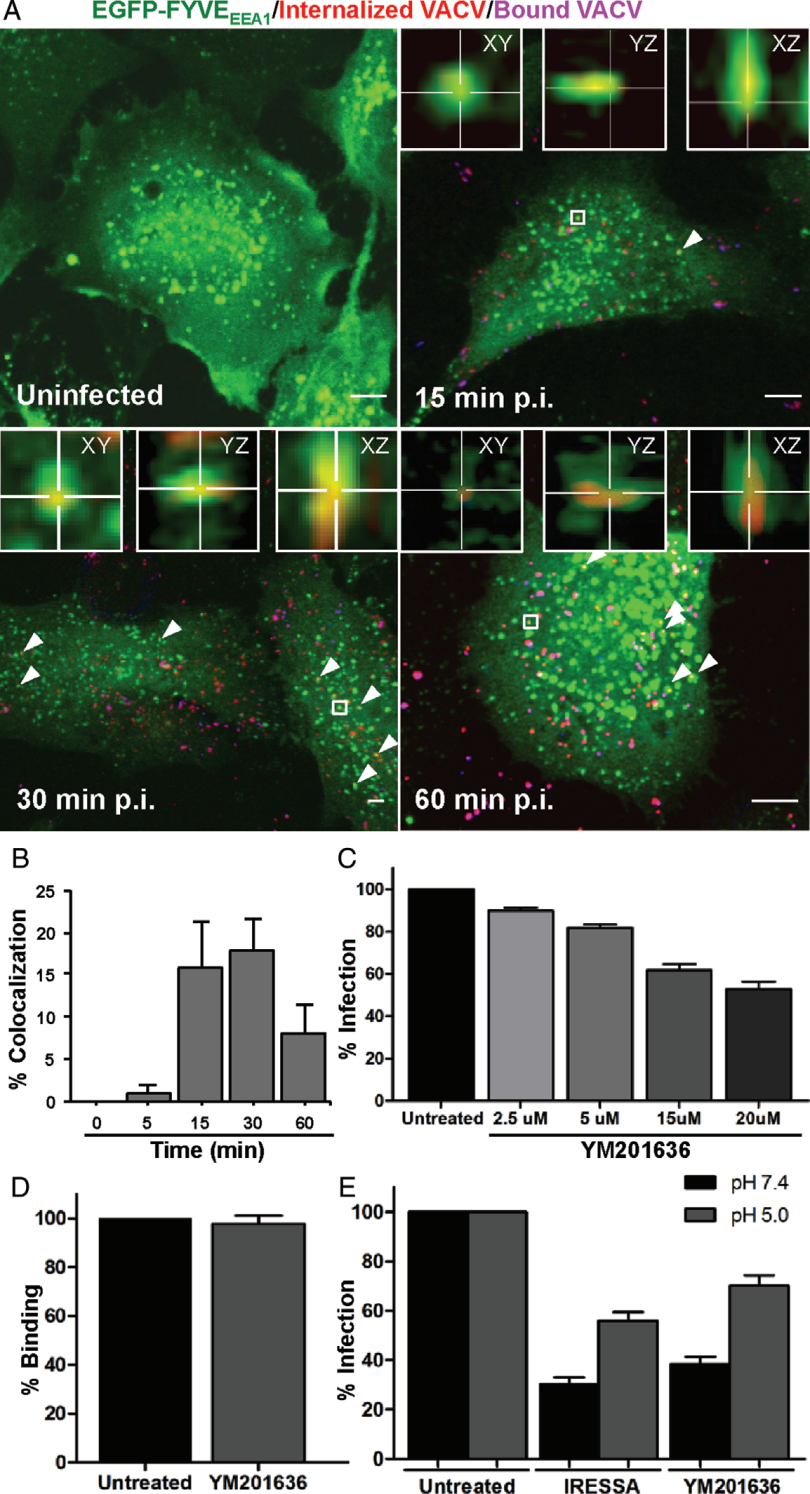
**PIKfyve** **activity is required for** **VACV MV** **entry.** A) HeLa cells transfected with EGFP‐FYVE_EEA1_ for 18 h were infected with WR mCherry‐A4 MVs at an MOI of 2. At the indicated time points, cells were fixed and non‐permeabilized cells subjected to immunostaining with α‐L1R to distinguish bound virions (purple). White arrows represent colocalization events and representative images of the peak time points of colocalization are displayed. Insets display individual colocalization examples from boxed regions in xy, yz and xz planes (imaris). Bars, 5 µm. B) The kinetics of internalized MV colocalization with EGFP‐FYVE_EEA1_ were quantified and displayed as percent colocalization at each time point. At least 45 cells were analyzed at all time points. Error bars represent the SD of three independent experiments. C) HeLa cells were pretreated with the PIKfyve inhibitor YM201636 for 1 h prior to infection with WR E EGFP at an MOI of 1 in the presence of the inhibitor. At 6 h p.i., cells were prepared for flow cytometry and 10 000 cells scored for infection. Results are means of three independent experiments ± SD. D) HeLa cells were left untreated or were pretreated with YM201636 for 1 h. After which, cells were cooled to 4°C and WR EGFP‐A4 MVs were added at an MOI of 10. After 1 h of binding, cells were harvested for flow cytometry and 10 000 cells scored for associated EGFP fluorescence. Results represent the mean of three independent experiments ± SD. E) HeLa cells were pretreated with IRESSA (40 µM) or YM201636 (20 µM) for 1 h. Cells were cooled to 4°C and WR E/L EGFP MVs (MOI = 1) were bound to cells for 1 h. After binding, cells were washed and treated with 37°C media (pH 7.4 or 5.0) for 5 min. Cells were washed and media containing the inhibitors was added. Cells were then incubated for an additional 4 h at 37°C prior to flow cytometry analysis of 10 000 cells per sample. Results represent means of three independent experiments ± SD.

PIs are essential for the progressive maturation of endosomes and macropinosomes 52, 53. When cells expressing the PtdIns(3)P probe EGFP‐FYVE_EEA1_ were infected with WR mCherry‐A4 MVs and analyzed 15 min after warming by confocal microscopy, about 16.0% of the viruses were located in PtdIns(3)P‐positive vacuoles (Figure 4A,B). In addition, we observed recruitment of the PtdIns(3)P‐binding probe to VACV MV‐containing macropinosomes by live‐cell imaging (Movie S7). Consistent with its role in maturation, the colocalization between VACV MVs and EGFP‐FYVE_EEA1_ dropped steadily after 30 min with <8.0% of virions localizing with PtdIns(3)P‐positive compartments at 60 min (Figure 4A,B). These findings suggested that the VACV‐containing macropinosomes acquired PtdIns(3)P at early stages of virion trafficking which is then lost as the macropinosomes mature.

Our siRNA screen revealed that PIK3R4, the regulatory subunit of the PtdIns(3)P‐producing PIK3C3, as well as PIKfyve were required for VACV infection (Figure 2). PIKfyve catalyzes the conversion of PtdIns(3)P to PtdIns(3,5)P_2_, a process of importance for the maturation of endosomes and macropinosomes 3, 30, 54. To determine if PIKfyve‐mediated conversion of PtdIns(3)P to PtdIns(3,5)P_2_ was important for VACV infection, cells were infected with WR E EGFP MVs in the presence of increasing concentrations of YM201636, an inhibitor of PIKfyve. When analyzed by flow cytometry 6 h p.i., a dose‐dependent inhibition of VACV MV infection, up to 50%, was observed (Figure 4C).

To ensure that YM201636 prevented infection at the level of virus entry, virus binding and macropinocytosis bypass assays were performed. WR EGFP‐A4 MVs were first allowed to bind to cells on ice in the absence or presence of YM201636. When cell‐associated fluorescence was quantified by flow cytometry as described 55, no defect in virus binding to cells was observed (Figure 4D).

Taking advantage of the acid‐mediated fusion activity of VACV MVs 46, virus penetration can be forced at the plasma membrane by low pH, thus bypassing endocytosis. We have previously employed this strategy to test the specificity of entry inhibitors 34, 55. To determine whether YM201636 inhibited MV entry, WR E EGFP MVs were bound to cells in the presence or absence of YM201636 and subsequently treated with pH 5.0 media (Figure 4E). The epidermal growth factor inhibitor Iressa, previously shown to inhibit VACV infection at the level of entry 33, was used as a positive control for bypass. For both, exposure to pH 7.4 media served as a bypass control. In cells treated with YM201636 and low pH, VACV infection could be partially restored (Figure 4E). The nearly twofold rescue was significant (p = 0.0016) and comparable to that seen in control cells treated with Iressa and low pH (Figure 4E).

With the presence of PtdIns(3)P on MV‐containing macropinosomes and the requirement of PIKfyve activity for VACV entry, we concluded that PIKfyve mediate PI exchange and, by extension, PtdIns(3,5)P_2_ synthesis was needed for macropinosome maturation and productive entry of VACV MVs.

### VACV EV infection relies on macropinosome maturation factors

In addition to MVs, a second infectious form of virus is produced during the VACV life cycle known as EVs. EVs consist of an MV‐like particle surrounded by an additional Golgi‐derived membrane containing cellular and at least six additional viral proteins 56. We have previously demonstrated that similar to MVs, EVs enter HeLa cells by inducing their own macropinocytic uptake 55. To overcome the topological constraint of having two membranes, the outer EV membrane is shed in an acid‐dependent fashion within macropinosomes to free the underlying MV for fusion 36.

**Figure 5:**
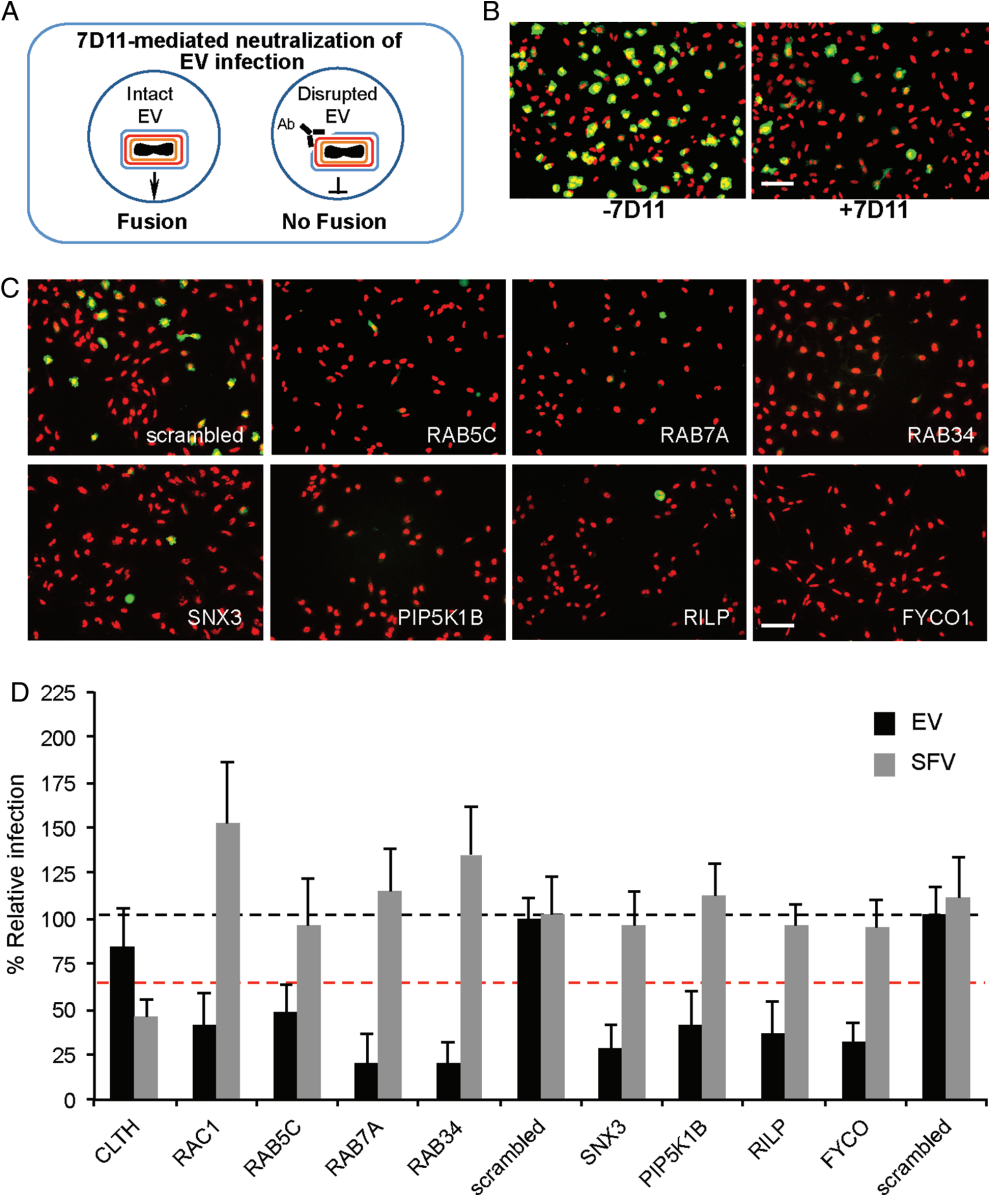
**VACV EVs** **have similar trafficking requirements as** **MVs**. A) Schematic representation of α‐L1R‐mediated neutralization of disrupted EVs. Neutralization of disrupted EVs targets the viral fusion machinery that is only present in the underlying MV membrane. B) WR E EGFP EVs collected from infected cell media were concentrated by centrifugation and subjected to α‐L1R neutralization. HeLa cells were then infected with non‐neutralized and α‐L1R neutralized EVs for comparison. Cells were stained with DAPI to visualize the nuclei (red) and the percentage of infected cells (green) determined. Scale bar, 50 µm. C) Representative images of hits from the EV RNAi screen, nuclei (red), infected cells (green). Scale bar, 50 µm. D) Comparison of the effects of the VACV EV hits on SFV infection. Depletion of clathrin heavy chain (CLTC) and Rac1 served as positive controls for inhibition of SFV and VACV EV infection, respectively. Results represent means of three independent experiments ± SD. Black dashed line is set at 100% relative to scrambled controls. Red dashed line is set at 40%, the cutoff for definition as a hit in the screen.

To determine if EVs share similar trafficking requirements as MVs, a subset of the LE factors required for MV trafficking were screened for their effect on EV infection (Table S2). We have previously shown that during preparation a large fraction of EVs are disrupted 36. To circumvent any consequences of disrupted EVs or contaminating MVs on investigation of EV entry, preparations of EVs were treated with the MAb 7D11. This antibody is directed against L1R, a component of the VACV fusion machinery, which is only present in the MV membrane. Thus, MVs and disrupted EVs are rendered fusion incompetent by 7D11 neutralization, while intact EVs remain unaffected (illustrated in Figure 5A).

For the screen, WR E EGFP EVs were prepared and subjected to 7D11 neutralization as previously described 36. Neutralization of EV preparations resulted in a threefold reduction (from 57 to 18%) in infection relative to untreated EV preparations (Figure 5B). The screen was performed in triplicate as described for Figure 2. Of the 10 factors assessed, depletion of 7 caused at least 40% reduction in infection with 2 or 3 of the 3 siRNAs and were defined as hits (Table S3). These include Rab5c, Rab7a, Rab34, SNX3, PIKP5K1B, RILP and FYCO (Figure 5C). Depletion of clathrin light chain (CLTC), required for SFV, or Rac1, required for VACV infection, again indicated that the inhibition of EV infection was not due to a general defect in the endosome system or cell fitness (Figure 5D). Collectively, these screening results indicated that for infectious entry both VACV MVs and EVs must traffic through macropinosomes that undergo maturation.

## Discussion

VACV MVs and EVs initiate entry into host cells by triggering their own macropinocytic uptake 34, 35, 37, 38. About 30 min later, the viral core is delivered into the cytosol after fusion of the viral envelope with the limiting membrane of endocytic vacuoles 39, 46. In this study, we followed the viruses during the time of passage within the endocytic network, and analyzed the maturation of the virus‐containing macropinosomes.

The first conversion that macropinosomes undergo after fission from the plasma membrane is the recruitment of EE factors such as Rab5 and its effector EEA1 as well as the accumulation of PtdIns(3)P 17, 18, 20. We observed MVs in Rab5a‐positive, and to a lesser extent in EEA1‐positive, vacuoles already 5 min after warming. The number within Rab5a‐positive vacuoles reached a maximum of 18% of internalized virions by 15 min. The association of macropinosomes with EEA1 was less prominent than with Rab5 reaching a maximum of 8.0%. That this Rab5 effector and tethering factor is only found on a subset of EEs 43, 57 may explain the low level of colocalization we observed. Perhaps, early macropinosomes, such as classical endosomes, exist as several distinct subsets.

Another possibility is that due to the asynchronous nature of VACV infection, combined with the transient role of EEA1 in promoting homotypic fusion of early macropinosomes, we only observe a fraction of actual virus EEA1 colocalization events 20, 22, 28. In fact, we could observe multiple rapid fusion events between virus‐containing, Rab5a‐positive early macropinosomes by live‐cell imaging (Movies S1 and S2). It is possible that the fusion events also involved one of the hits in our RNAi screen, vesicle‐associated membrane protein 7 (VAMP7). It has been shown to mediate pinosome fusion events in *Dictyostelium discoideum* 58.

Most of the early vacuoles were positive for PtdIns(3)P. We could follow the generation of this lipid in virus‐containing vacuoles by live‐cell imaging (Movie S7). We and others have previously shown that the vacuoles containing MVs also contain fluid markers such as dextran, and that they are devoid of transferrin, a cellular cargo molecule present in early and recycling endosomes 34, 35, 37, 38. This is consistent with reports indicating that classical endosomes and macropinosomes give rise to distinct transport vesicle populations 28.

In addition to homotypic fusion, macropinosomes undergo tubulation mediated by SNX family members 22. Some SNXs have also been reported to facilitate macropinosome maturation and function 23, 25, 59. Having identified SNX3 as a hit in the RNAi screen, we investigated its localization in cells that had internalized VACV MVs. SNX3 was predominantly found in MV‐containing LMs (Figure S4). Interestingly, SNX3 has been shown to localize to SCVs where it facilitates SCV maturation to late compartments 25.

Maturation of virus‐containing vacuoles continued about 15 min after warming with Rab5a being gradually replaced by Rab7a. LAMP1, a membrane glycoprotein found on LE and LYs, arrived shortly after this conversion. Vacuoles positive for both Rab7a and LAMP1 are known to serve as terminal organelles during trafficking of the fluid phase marker dextran 60. The change in Rabs and other markers thus suggested that VACV‐containing macropinosomes underwent a full program of maturation, in line with the relatively late timing of VACV MV penetration. The VACV strain used here, WR, has been shown to require low pH (pH = 4.5–5.0) for envelope fusion 46, 61, 62. However, inhibition of WR MV infection by lysosomotropic agents varies within the range of 40–60%, and other VACV strains, such as IHD‐J, fuse from macropinosomes in a pH‐independent fashion 33, 44. This suggests that other macropinosome factors, in addition to low pH, may be required for VACV fusion. It is also possible that the acidification of macropinosomes or their sensitivity to lysosomotropic agents is not identical to that of canonical endosomes.

Rab7a depletion and the expression of either D/N or C/A mutants inhibited MV infection. The Rab7a effectors identified as hits in the RNAi screen, RILP, FYCO1 and OSBPL1A, are all involved in endosome movement. FYCO1 interacts with kinesins, and OSBPL1A promotes binding of RILP to dynein to mediate the movement of Rab7‐positive vesicles along microtubules (MTs) 63, 64. Another hit, Rab34, confirmed by D/N and C/A expression (Figure S5) has been implicated in regulation of lysosomal positioning through interaction with RILP 65.

The involvement of these factors implied that MV‐containing macropinosomes interact with MT‐mediated motors for transport of the vacuoles toward the perinuclear region of the cell where the majority of LYs are located 3. However, as previously reported 39, neither depolymerization of MTs with nocodazole or colchicine, nor stabilization with taxol had any effect on VACV early infection events (Figure S6). Although in agreement with the co‐existence of MT‐dependent and ‐independent macropinocytic pathways 66, in light of these findings we suspect that MT‐based transport of VACV‐containing macropinosomes is not necessary for VACV infection, but rather serves to increase the likelihood of macropinosome fusion with LEs or LYs, which could serve to speed entry kinetics.

PI kinases also play a central role in regulating the maturation of endosomes, phagosomes and macropinosomes 67. Our RNAi screen indicated that PI(3)K, PI(4)K, PI(5)K and PIKfyve were needed for MV infection (Figure 2). Previous studies have demonstrated a role for PI(3)K during multiple stages of VACV infection 68, 69 including entry 34, 39, 70. Consistent with this, PtdIns(3)P was detected on early MV‐containing macropinosomes until 30 min suggesting that these compartments undergo a lipid switch during their maturation (Figure 4A,B).

The identification of PIKfyve as a hit in the RNAi screen and the inhibitory effect of a PIKfyve inhibitor, YM201636, indicated that conversion of PtdIns(3)P to PtdIns(3,5)P_2_ is critical for VACV MV infection. PIKfyve facilitates Rab7a‐independent fusion of LEs with each other and with LYs 30. In addition, the PIKfyve‐activating kinase, Akt, previously implicated in VACV entry 70, 71, was a hit in our screen. Interestingly, Akt has been reported to phosphorylate PIKfyve to facilitate lysosomal trafficking and degradation of EGFR 72.

VACV EVs also enter cells by macropinocytosis but the process of penetration is more complicated due to the additional envelope membrane 35, 37. That the hits were largely overlapping with VACV MVs strongly suggested that EVs also require macropinosome maturation for productive infection. As EVs shed their outermost membrane within macropinosomes 35, and rely on the underlying low pH‐dependent MV fusion machinery 46, 61, 62, 73, it is not too surprising that they are subject to the same requirements as MVs.

**Figure 6:**
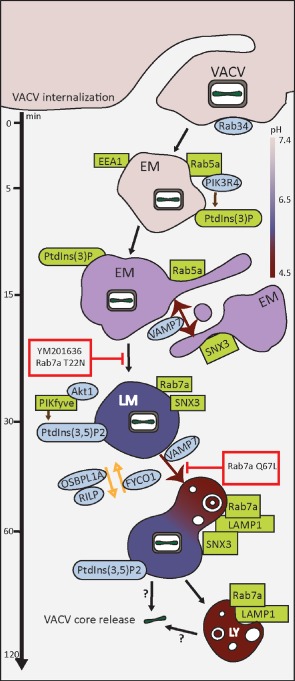
**Model of** **VACV** **macropinocytic trafficking.** VACV enters host cells by macropinocytosis. Within 5 min p.i., VACV MVs can be visualized in early macropinosomes (EM) positive for Rab5a, EEA1 and PtdIns(3)P. Localization of MVs with these early macropinosomes (EMs) peaked 15 min p.i. At this time, equivalent numbers of MVs were found in Rab5a‐positive EMs and Rab7a‐positive late macropinosome (LMs). This suggests that the Rab conversion occurred at this time point. By 30 min p.i., the majority of internalized MVs were found in Rab7a‐positive LMs which began to acquire LAMP1. The number of MVs in Rab7a‐ and LAMP1‐positive LMs was equivalent by 60 min p.i. Colocalization of MVs with LMs dropped steadily over the next 60 min consistent with the majority of virions fusing from Rab7a/LAMP1‐positive compartments. Whether these represent LMs or lysosomes (LYs) remains to be determined. Relevant trafficking factors identified in the RNAi screen and confirmed by localization, live‐cell imaging or perturbation analysis are in green. Additional endocytosis factors identified in the RNAi screen as required for VACV infection are in blue. The stage of VACV MV trafficking inhibited by Rab7a mutants and PIKfyve inhibitor, YM 201636, are displayed in red boxes.

Viruses provide many advantages as tools to characterize the complex conversions that take place before cargo‐carrying vacuoles fuse with LYs. Using VACV as ligand, we found that the process of macropinosome maturation shares many features with the maturation of endosomes 3, phagosomes 74 and autophagosomes 75. Our findings indicated that maturation was essential for the infectivity of VACV MVs and EVs (Figure 6). It was evident that VACV penetration occurred from macropinosomes quite late in the maturation program. This was illustrated by the finding that PIKfyve and Akt were needed, that delivery to Rab7a‐positive compartments was necessary but not sufficient for infection and is consistent with the pH optimum of VACV fusion which is as low as 4.5 46, 61. It is even conceivable that fusion with LYs is required for productive infection. Collectively, our findings support the classification of VACV as a late‐penetrating virus 14, and provide new cellular targets for the development of antiviral agents against viruses that use macropinocytosis as an entry route.

## Materials and Methods

### Cells and viruses

HeLa ATCC cells were propagated in DMEM including 10% fetal calf serum (FCS), non‐essential amino acids and Glutamax (Life technologies). Recombinant VACVs used in the study were generated using the VACV WR strain as previously described 34. These include fluorescent expressors WR E EGFP and WR E/L EGFP as well as viruses containing fluorescent proteins fused to the N‐terminus of the structural core protein A4 (WR EGFP‐A4 and WR mCherry‐A4).

### Plasmids and plasmid transfections

EGFP‐Rab5a, EGFP‐Rab7a, EGFP‐Rab7a Q67L, EGFP‐Rab7a T22N and LAMP1‐EGFP plasmids were previously described 76. RFP‐Rab34, RFP‐Rab34 Q111L and RFP‐Rab34 T66N were kindly provided by U. Greber (University of Zurich). EGFP‐FYVE_EEA1_ was kindly provided by S. Grinstein (University of Toronto). EGFP‐SNX1 was kindly provided by R. Teasdale (University of Queensland). EGFP‐SNX3 was kindly provided by J. Gruenberg (University of Geneva). Cells were transfected with plasmids using Lipofectamine 2000 (Invitrogen) or the Amaxa cell line Nucleofector kit R (Lonza) according to the manufacturer's instructions.

### Antibodies and inhibitors

Antibodies directed against EEA1 and mRFP were purchased from Cell Signaling and Clontech, respectively. The mouse MAb 7D11 (α‐L1) was kindly provided by Bernard Moss with permission of Alan Schmaljohn (University of Maryland). Secondary antibodies were obtained from Invitrogen. Stocks of YM201636 (Calbiochem), IRESSA (LC Laboratories), Taxol, Colchicine and Nocodazole (Sigma) were prepared in dimethyl sulfoxide and stored at −20°C.

### Colocalization assays

Cells were transfected with plasmids on coverslips in 24‐well plates and infected with VACV mCherry‐A4 (multiplicity of infection, MOI = 20) in FCS‐free medium. Virus was bound to cells for 30 min in a cold water bath. Cells were then washed and incubated in a 37°C water bath for indicated time points (up to 4 h). Bound non‐internalized particles were stained with the MAb 7D11 on ice without permeabilization. Fixed cells were analyzed with a Zeiss LSM 510 Meta confocal microscope using a 63× objective and seven to ten Z‐stacks were acquired per image. All images represent maximum projects when not indicated otherwise. Cells analyzed for endogenous markers were first subjected to 7D11 staining as above, fixed and permeabilized for 2 min with 0.1% Triton‐X‐100 prior to staining with antibodies directed against each marker, followed by Alexa Fluor 488 secondary antibody. Antibodies used were Rab5 [Cell Signaling; Rab5 (C8B1)], Rab7 [Cell Signaling; Rab7 (D95F2)], LAMP1 [(Abcam; LAMP1 (ab24170)] and EEA1 [Cell Signaling; EEA1 (3288)].

### Quantification of colocalization of internalized VACV with endosomal markers

imaris software was used to automatically quantify the fraction of internalized virions that colocalize with the various endocytic markers (Rab5, EEA1, Rab7, LAMP1 and FYVE‐EGFP) (Figure S1). For each three‐dimensional confocal stack, virus particles and endocytic vesicles were automatically detected using the imaris spot colocalization tool. For each image, the thresholding value was set to 200 nm to ensure maximum accuracy of particle detection. Internalized viruses were defined as mCherry‐positive virions that did not colocalize with VACV particles positive for 7D11 immunostaining (i.e. non‐internalized viruses). For this, mCherry viruses were considered as colocalizing if their median fluorescence was equal to or above the threshold used to identify immunostained particles. The percentage of internalized virions was then determined by dividing the number of internalized virions by the total virion count at each time point assayed. The external virions were not considered for the downstream colocalization analyses. Using a similar procedure, the remaining internalized viral particles were assessed for colocalization with the various endocytic markers. Virions were considered to colocalize only if the viral particle showed a 100% overlap with the various endosomal marker proteins. Manual correction was employed in instances of high local background to prevent colocalization errors. Colocalization was scored in at least 45 cells per time point. The average percent colocalization of internalized MVs with each marker is displayed for each time point. Error bars represent the SD.

Live cells were analyzed with an Olympus Cell^R epifluorescence microscope in eight‐well chamber slides using a 100× oil objective. For live‐cell imaging, binding and infection were performed as described above and images were captured at various time points post‐infection at 0.5 Hz for up to 5 min.

### Inhibitor studies

HeLa cells were seeded in 24‐well plates and pre‐treated with different concentrations of YM201636 for 1 h. Cells were then infected with WR E EGFP (MOI = 1) in the presence of the inhibitor. At 6 h p.i., samples were harvested, prepared for flow cytometry and the percentage of infected cells was determined on the basis of EGFP fluorescence.

### Rab mutants

HeLa cells were seeded in six‐well plates and transfected with plasmids expressing EGFP‐tagged WT Rab7a and the respective C/A and D/N variants. After 18 h, cells were infected with WR E/L mRFP (MOI = 1). Cells were harvested 6 h p.i. and scored for both EGFP and mRFP expression by flow cytometry. For microscopy, cells were transfected and prepared as described above for colocalization assays.

### Binding assay

HeLa cells were seeded in 24‐well plates and pre‐treated with YM201636 (20 µμ) for 1 h. Cells were then infected with WR EGFP‐A4 (MOI = 10). Virus was allowed to bind to cells at 4°C. Cells were then washed several times and prepared for fluorescence‐activated cell sorter (FACS) analysis in order to quantify virus binding based on cell‐associated EGFP fluorescence intensity.

### Acid bypass assay

HeLa cells seeded in 24‐well plates were pre‐treated with IRESSA (40 µµ) or YM201636 (20 µμ) for 1 h. WR E/L EGFP (MOI = 1) was allowed to bind to the cell surface by incubating the plate at 4°C for 1 h. DMEM was adjusted to pH 5.0 with 20 mm 2‐(N‐morpholino)ethanesulfonic acid. After binding, cells were washed, 500 µL of 37°C infection medium (pH 5.0 or 7.4) was added to separate wells and the plate was transferred into a 37°C water bath for 5 min to allow fusion of the virus with the plasma membrane. Samples were washed with warm medium and 500 µL of DMEM (+) containing the appropriate inhibitor was added to each well. The plate was incubated at 37°C for 4 h and prepared for flow cytometry.

For all flow cytometry‐based assays, cells were washed once in PBS followed by trypsinization from the assay plate. Trypsin was neutralized with fetal calf serum and formaldehyde was added to 4% final concentration. After 15 min, cells were pelleted, washed once in PBS and resuspended in PBS/ethylenediaminetetraacetic acid for flow cytometry. Following preparation, cells were analyzed using a BD FACSCalibur flow cytometer (Becton Dickinson) and the flow jo software package (Treestar).

### siRNA screen

For reverse transfection of siRNA, Lipofectamine RNAi Max (Life technologies) transfection reagent was mixed with serum‐free DMEM. Then, 25 µL of the transfection mix was dispensed into each well of a 384‐well plate containing 5 µL (320 nm) of each individual target siRNAs. Plates were incubated for 1 h at room temperature prior to the addition of 600 HeLa cells to each well dispensed in 50 µL of DMEM containing 16% FCS. Plates were then incubated at 37°C for 72 h.

Cells were infected with WR E EGFP to achieve an infection index of 20%. Cells were incubated with the virus for 1 h at 37°C, washed and 80 µL of growth media was added per well. The plates were incubated for 6 h at 37°C. Subsequently, the cells were washed and fixed with 4% formaldehyde. For EVs, BSC40 cells were infected 24 h prior to screening and α‐L1R‐neutralized EVs were prepared as previously described 35. Neutralized EV preparations were then used for infection.

To boost the E EGFP signal for imaging, cells were subjected to immunostaining with a rabbit polyclonal clonal α‐EGFP antibody (1:1000), followed by Alexafluor‐488 coupled α‐rabbit secondary antibody (1:1000) (Invitrogen), and Hoechst (1 µg/mL) for nuclear staining. Plates were imaged on an imagexpress Microscreening system (Molecular Device). Image analysis was performed with matlab 48 and is described in Figure S3.

## Supporting information


**Figure S1: Automated analysis of VACV internalization and endosome colocalization.** A) Detection of endocytic vesicles (endosomes) and viral particles using the imaris software. Digital images of endocytic vesicles, total virus particles (VACV tot) and external virus particles (VACV ext) generated using the imaris spot detection are displayed. B) Automatic detection of internalized virions (VACV int) with imaris. For this, internalized mCherry‐positive virus particles were defined as those that have no associated VACV ext fluorescence. C) The imaris ‘spot colocalization’ tool was used to automatically detect the colocalization between internalized virions (VACV int) and endocytic vesicles (endosomes). A virion and an endocytic vesicle were considered to colocalize when their distance (from the center or each spot) was ≤200 nm. White arrowheads indicate internalized virus particles that colocalize with endosomal vesicles. Non‐internalized virions (VACV ext) were used as negative controls (light‐blue arrowhead).

**Figure S2: Colocalization of VACV with endogenous Rab5, Rab7 and LAMP1.** A–C) HeLa cells were bound with VACV WR mCherry‐A4 MVs at an MOI of 2 at 4°C. Cells were washed and shifted to 37°C for the indicated time points. Non‐permeabilized cells were then subjected to immunostaining with α‐L1R to distinguish external (blue) versus internalized (red) virions. To visualize endogenous Rab5 (A), Rab7 (B) or LAMP1 (C), cells were permeabilized and immunostained using antibodies directed against these various markers. Insets display colocalization events in the xy, yz and xz planes. White arrows represent colocalization events. D) The percent colocalization between internalized virions and the various endocytic markers was determine using imaris automated colocalization analysis as described in Figure S1. At least 30 total cells from three independent experiments were analyzed for each marker. Results displayed as the average ± SD.

**Figure S3: RNAi** **screen workflow, image analysis and cell number correction**. A) The ‘usual suspects’ siRNA library consists of two 384‐well plates. Three copies of the library (six plates in total) were used in each experiment. The siRNAs were introduced into HeLa ATCC cells by reverse transfection. At 72 h post‐transfection, cells were infected with WR E EGFP MVs. At 6 h p.i., cells were fixed, nuclei stained with DAPI and the EGFP signal was enhanced by immunofluorescence staining using an α‐EGFP antibody. Assay plates were then imaged using an image xpress Microscreening system. The screen was repeated three independent times and the results were shown as the mean of the triplicates. B) Image analysis was performed using an in‐house matlab‐based software that allowed for automatic digital detection and scoring of nuclei and EGFP‐positive infected cells (scale bars, 50 µm). C) To correct for the effect on infection index due to deleterious effects of RNAi transfection on cell number variability, an infection index checkerboard was used for correction. Infection of a gradient of cells treated with control siRNA (AllStarNegative) was used to determine the correlation between the number of cells and the corresponding infection index. This was then used to create a normalization curve that was applied to the screening data to eliminate any cell number bias on infection. Any siRNA target wells displaying <200 cells were discarded from the analysis.

**Figure S4: Colocalization of VACV MVs with SNX3.** Cells transfected with EGFP‐SNX3 were infected with WR mCherry‐A4 MVs at an MOI of 2. At the indicated time points, cells were fixed and non‐permeabilized cells were subjected to immunostaining with α‐L1R to distinguish bound virions (purple). White arrows represent colocalization events and representative images of the peak time points of colocalization are displayed. Insets display individual colocalization examples from boxed regions in xy, yz and xz planes (imaris). Bars, 5 µm.

**Figure S5: VACV** **MV infection relies on Rab34 function.** HeLa cells were transfected with WT, C/A or D/N versions of EGFP‐Rab34. At 18 h p.i., cells were infected with WR E/L mRFP MVs. Cells were harvested for flow cytometry, and 10 000 transfected cells were scored for infection. Results are displayed as the percent infection relative to infection of WT Rab34 overexpressing cells and represent the means of three independent experiments ± SD.

**Figure S6: VACV** **MV infection does not require MT dynamics.** HeLa cells were pre‐treated with the indicated compounds at 10 µm for 1 h prior to infection. Cells were then infected with WR E EGFP L mCherry virus (MOI = 2). At 12 h p.i., cells were harvested and analyzed by flow cytometry for both EGFP (black bars; early gene expression) and mCherry (gray bars; late gene expression). The average of two independent experiments is displayed as percent infection relative to control infections set at 100%.

**Table S1:** The ‘usual suspects’ siRNA library. Listed are the three independent siRNAs used for depletion of 162 human genes involved in endocytosis and membrane trafficking. Information includes Entrez gene id (column A), NCBI gene symbol (column B), gene description (column C) and siRNA target sequence (column E).

**Table S2:** VACV MV trafficking hits. The thirty‐three cell factors whose depletion caused at least a 40% decrease in MV infection are listed. For each siRNA used, the Entrez Gene Id (column A), NCBI gene symbol (column B), average relative infection (column H) and average number of nuclei (column J) are displayed.

**Table S3:** Trafficking hits required for EV infection. Listed are the 10 MV hits (see Table S2) whose depletion resulted in a 40% or greater decrease in EV infection. For each siRNA, the Entrez gene Id (column A), NCBI gene symbol (column B), average relative infection (column H) and average number of nuclei (column J) are displayed.

**Movie S1.** Dynamic colocalization of VACV MVs with Rab5a. HeLa cells expressing EGFP‐Rab5a were infected with WR mCherry‐A4 MVs. Cells were imaged 15 min p.i. at 0.5 Hz. Note coordinated dynamic movement of VACV MVs and Rab5a. Regions of interest are boxed in white.

**Movie S2.** Dynamic colocalization of VACV MVs with Rab5a. HeLa cells expressing EGFP‐Rab5a were infected with WR mCherry‐A4 MVs, and cells imaged 15 min. p.i. at 0.5 Hz. Note the fusion of VACV MV containing early macropinosomes with large Rab5a positive vacuoles.

**Movie S3.** Dynamic colocalization of VACV MVs with Rab7a‐positive late macropinosomes. HeLa cells expressing EGFP‐Rab7a were infected with WR mCherry‐A4 MVs. Cells were imaged 30 min p.i. at 0.5 Hz. Note the large size of the macropinosome in Movie S3 which appears to contain multiple MVs. Regions of interest are boxed in white.

**Movie S4.** Dynamic colocalization of VACV MVs with Rab7a‐positive late macropinosomes. HeLa cells expressing EGFP‐Rab7a were infected with WR mCherry‐A4 MVs, and cells imaged 30 min. p.i. at 0.5 Hz. Note the fusion of VACV MV containing late macropinosomes with large Rab7a positive vacuoles.

**Movie S5.** Dynamic colocalization of VACV MVs with LAMP1. HeLa cells expressing LAMP1‐EGFP were infected with WR mCherry‐A4 MVs and cells were imaged 30 min p.i. at 0.5 Hz. Note the large size and highly dynamic movement of the LAMP1‐positive compartments. A VACV MV fusing from a LAMP1‐positive late macropinosome can be seen in Movie S5 (center box). Regions of interest are boxed in white.

**Movie S6.** Dynamic colocalization of VACV MVs with LAMP1. HeLa cells expressing LAMP1‐EGFP were infected with WR mCherry‐A4 MVs, and cells imaged 30 min. p.i. at 0.5 Hz. Note the fusion of VACV MV containing LAMP1‐positive vacuoles.

**Movie S7.** Dynamic colocalization of VACV MVs with EGFP‐FYVE_EEA1_. HeLa cells expressing EGFP‐FYVE_EEA1_ were infected with WR mCherry‐A4 MVs and cells were imaged 15 min p.i. at 0.5 Hz. Note the acquisition of EGFP‐FYVE_EEA1_ on MVs and their subsequent movement within EGFP‐FYVE_EEA1_‐positive compartments. Regions of interest are boxed in white.

## References

[1] Marsh M , Helenius A . Virus entry: open sesame. Cell 2006;124:729–740. 16497584 10.1016/j.cell.2006.02.007 PMC7112260

[2] Mercer J , Schelhaas M , Helenius A . Virus entry by endocytosis. Annu Rev Biochem 2010;79:803–833. 20196649 10.1146/annurev-biochem-060208-104626

[3] Huotari J , Helenius A . Endosome maturation. EMBO J 2011;30:3481–3500. 21878991 10.1038/emboj.2011.286 PMC3181477

[4] Scott CC , Vacca F , Gruenberg J . Endosome maturation, transport and functions. Semin Cell Dev Biol 2014;31:2–10. 24709024 10.1016/j.semcdb.2014.03.034

[5] Poteryaev D , Datta S , Ackema K , Zerial M , Spang A . Identification of the switch in early‐to‐late endosome transition. Cell 2010;141:497–508. 20434987 10.1016/j.cell.2010.03.011

[6] Solinger JA , Spang A . Tethering complexes in the endocytic pathway: CORVET and HOPS. FEBS J 2013;280:2743–2757. 23351085 10.1111/febs.12151

[7] Woodman PG . Biogenesis of the sorting endosome: the role of Rab5. Traffic 2000;1:695–701. 11208157 10.1034/j.1600-0854.2000.010902.x

[8] Jovic M , Sharma M , Rahajeng J , Caplan S . The early endosome: a busy sorting station for proteins at the crossroads. Histol Histopathol 2010;25:99–112. 19924646 10.14670/hh-25.99 PMC2810677

[9] Gillooly DJ , Morrow IC , Lindsay M , Gould R , Bryant NJ , Gaullier JM , Parton RG , Stenmark H . Localization of phosphatidylinositol 3‐phosphate in yeast and mammalian cells. EMBO J 2000;19:4577–4588. 10970851 10.1093/emboj/19.17.4577 PMC302054

[10] Odorizzi G , Babst M , Emr SD . Fab1p PtdIns(3)P 5‐kinase function essential for protein sorting in the multivesicular body. Cell 1998;95:847–858. 9865702 10.1016/s0092-8674(00)81707-9

[11] Maxfield FR , Yamashiro DJ . Endosome acidification and the pathways of receptor‐mediated endocytosis. Adv Exp Med Biol 1987;225:189–198. 2839960 10.1007/978-1-4684-5442-0_16

[12] Pasqual G , Rojek JM , Masin M , Chatton JY , Kunz S . Old world arenaviruses enter the host cell via the multivesicular body and depend on the endosomal sorting complex required for transport. PLoS Pathog 2011;7:e1002232. 21931550 10.1371/journal.ppat.1002232 PMC3169553

[13] Lozach PY , Mancini R , Bitto D , Meier R , Oestereich L , Overby AK , Pettersson RF , Helenius A . Entry of bunyaviruses into mammalian cells. Cell Host Microbe 2010;7:488–499. 20542252 10.1016/j.chom.2010.05.007 PMC7172475

[14] Lozach PY , Huotari J , Helenius A . Late‐penetrating viruses. Curr Opin Virol 2011;1:35–43. 22440565 10.1016/j.coviro.2011.05.004

[15] Mercer J , Helenius A . Virus entry by macropinocytosis. Nat Cell Biol 2009;11:510–520. 19404330 10.1038/ncb0509-510

[16] Swanson JA . Shaping cups into phagosomes and macropinosomes. Nat Rev Mol Cell Biol 2008;9:639–649. 18612320 10.1038/nrm2447 PMC2851551

[17] Feliciano WD , Yoshida S , Straight SW , Swanson JA . Coordination of the Rab5 cycle on macropinosomes. Traffic 2011;12:1911–1922. 21910808 10.1111/j.1600-0854.2011.01280.x PMC3213305

[18] Schnatwinkel C , Christoforidis S , Lindsay MR , Uttenweiler‐Joseph S , Wilm M , Parton RG , Zerial M . The Rab5 effector Rabankyrin‐5 regulates and coordinates different endocytic mechanisms. PLoS Biol 2004;2:E261. 15328530 10.1371/journal.pbio.0020261 PMC514490

[19] Sun P , Yamamoto H , Suetsugu S , Miki H , Takenawa T , Endo T . Small GTPase Rah/Rab34 is associated with membrane ruffles and macropinosomes and promotes macropinosome formation. J Biol Chem 2003;278:4063–4071. 12446704 10.1074/jbc.M208699200

[20] Hamasaki M , Araki N , Hatae T . Association of early endosomal autoantigen 1 with macropinocytosis in EGF‐stimulated A431 cells. Anat Rec 2004;277:298–306. 10.1002/ar.a.20027 15052657

[21] Bryant DM , Kerr MC , Hammond LA , Joseph SR , Mostov KE , Teasdale RD , Stow JL . EGF induces macropinocytosis and SNX1‐modulated recycling of E‐cadherin. J Cell Sci 2007;120:1818–1828. 17502486 10.1242/jcs.000653

[22] Kerr MC , Lindsay MR , Luetterforst R , Hamilton N , Simpson F , Parton RG , Gleeson PA , Teasdale RD . Visualisation of macropinosome maturation by the recruitment of sorting nexins. J Cell Sci 2006;119:3967–3980. 16968745 10.1242/jcs.03167

[23] Lim JP , Wang JT , Kerr MC , Teasdale RD , Gleeson PA . A role for SNX5 in the regulation of macropinocytosis. BMC Cell Biol 2008;9:58. 18854019 10.1186/1471-2121-9-58 PMC2576169

[24] Merino‐Trigo A , Kerr MC , Houghton F , Lindberg A , Mitchell C , Teasdale RD , Gleeson PA . Sorting nexin 5 is localized to a subdomain of the early endosomes and is recruited to the plasma membrane following EGF stimulation. J Cell Sci 2004;117:6413–6424. 15561769 10.1242/jcs.01561

[25] Braun V , Wong A , Landekic M , Hong WJ , Grinstein S , Brumell JH . Sorting nexin 3 (SNX3) is a component of a tubular endosomal network induced by Salmonella and involved in maturation of the Salmonella‐containing vacuole. Cell Microbiol 2010;12:1352–1367. 20482551 10.1111/j.1462-5822.2010.01476.x

[26] Racoosin EL , Swanson JA . Macropinosome maturation and fusion with tubular lysosomes in macrophages. J Cell Biol 1993;121:1011–1020. 8099075 10.1083/jcb.121.5.1011 PMC2119679

[27] Egami Y , Araki N . Dynamic changes in the spatiotemporal localization of Rab21 in live RAW264 cells during macropinocytosis. PLoS One 2009;4:e6689. 19693279 10.1371/journal.pone.0006689 PMC2726762

[28] Hewlett LJ , Prescott AR , Watts C . The coated pit and macropinocytic pathways serve distinct endosome populations. J Cell Biol 1994;124:689–703. 8120092 10.1083/jcb.124.5.689 PMC2119947

[29] West MA , Bretscher MS , Watts C . Distinct endocytotic pathways in epidermal growth factor‐stimulated human carcinoma A431 cells. J Cell Biol 1989;109:2731–2739. 2556406 10.1083/jcb.109.6.2731 PMC2115909

[30] Kerr MC , Wang JT , Castro NA , Hamilton NA , Town L , Brown DL , Meunier FA , Brown NF , Stow JL , Teasdale RD . Inhibition of the PtdIns(5) kinase PIKfyve disrupts intracellular replication of Salmonella . EMBO J 2010;29:1331–1347. 20300065 10.1038/emboj.2010.28 PMC2868569

[31] Mercer J , Helenius A . Gulping rather than sipping: macropinocytosis as a way of virus entry. Curr Opin Microbiol 2012;15:490–499. 22749376 10.1016/j.mib.2012.05.016

[32] Moss B . Poxviridae: the viruses and their replication In: Knipe DM, Howley PM, editors. Fields Virology. Philadelphia: Lippincott‐Raven; 2013, pp. 2129–2159.

[33] Mercer J , Knebel S , Schmidt FI , Crouse J , Burkard C , Helenius A . Vaccinia virus strains use distinct forms of macropinocytosis for host‐cell entry. Proc Natl Acad Sci U S A 2010;107:9346–9351. 20439710 10.1073/pnas.1004618107 PMC2889119

[34] Mercer J , Helenius A . Vaccinia virus uses macropinocytosis and apoptotic mimicry to enter host cells. Science 2008;320:531–535. 18436786 10.1126/science.1155164

[35] Schmidt FI , Bleck CK , Helenius A , Mercer J . Vaccinia extracellular virions enter cells by macropinocytosis and acid‐activated membrane rupture. EMBO J 2011;30:3647–3661. 21792173 10.1038/emboj.2011.245 PMC3181475

[36] Schmidt FI , Bleck CK , Mercer J . Poxvirus host cell entry. Curr Opin Virol 2012;2:20–27. 22440962 10.1016/j.coviro.2011.11.007

[37] Sandgren KJ , Wilkinson J , Miranda‐Saksena M , McInerney GM , Byth‐Wilson K , Robinson PJ , Cunningham AL . A differential role for macropinocytosis in mediating entry of the two forms of vaccinia virus into dendritic cells. PLoS Pathog 2010;6:e1000866. 20421949 10.1371/journal.ppat.1000866 PMC2858709

[38] Huang CY , Lu TY , Bair CH , Chang YS , Jwo JK , Chang W . A novel cellular protein, VPEF, facilitates vaccinia virus penetration into HeLa cells through fluid phase endocytosis. J Virol 2008;82:7988–7999. 18550675 10.1128/JVI.00894-08 PMC2519564

[39] Locker JK , Kuehn A , Schleich S , Rutter G , Hohenberg H , Wepf R , Griffiths G . Entry of the two infectious forms of vaccinia virus at the plasma membrane is signaling‐dependent for the IMV but not the EEV. Mol Biol Cell 2000;11:2497–2511. 10888684 10.1091/mbc.11.7.2497 PMC14935

[40] Kilcher S , Schmidt FI , Schneider C , Kopf M , Helenius A , Mercer J . siRNA screen of early poxvirus genes identifies the AAA+ ATPase D5 as the virus genome‐uncoating factor. Cell Host Microbe 2014;15:103–112. 24439902 10.1016/j.chom.2013.12.008

[41] Dales S . The uptake and development of vaccinia virus in strain L cells followed with labeled viral deoxyribonucleic acid. J Cell Biol 1963;18:51–72. 14024720 10.1083/jcb.18.1.51 PMC2106286

[42] Mercer J , Snijder B , Sacher R , Burkard C , Bleck CK , Stahlberg H , Pelkmans L , Helenius A . RNAi screening reveals proteasome‐ and Cullin3‐dependent stages in vaccinia virus infection. Cell Rep 2012;2:1036–1047. 23084750 10.1016/j.celrep.2012.09.003

[43] Miaczynska M , Christoforidis S , Giner A , Shevchenko A , Uttenweiler‐Joseph S , Habermann B , Wilm M , Parton RG , Zerial M . APPL proteins link Rab5 to nuclear signal transduction via an endosomal compartment. Cell 2004;116:445–456. 15016378 10.1016/s0092-8674(04)00117-5

[44] Bengali Z , Townsley AC , Moss B . Vaccinia virus strain differences in cell attachment and entry. Virology 2009;389:132–140. 19428041 10.1016/j.virol.2009.04.012 PMC2700833

[45] Townsley AC , Moss B . Two distinct low‐pH steps promote entry of vaccinia virus. J Virol 2007;81:8613–8620. 17553884 10.1128/JVI.00606-07 PMC1951335

[46] Townsley AC , Weisberg AS , Wagenaar TR , Moss B . Vaccinia virus entry into cells via a low‐pH‐dependent endosomal pathway. J Virol 2006;80:8899–8908. 16940502 10.1128/JVI.01053-06 PMC1563910

[47] Whitbeck JC , Foo CH , Ponce de Leon M , Eisenberg RJ , Cohen GH . Vaccinia virus exhibits cell‐type‐dependent entry characteristics. Virology 2009;385:383–391. 19162290 10.1016/j.virol.2008.12.029 PMC4041486

[48] Engel S , Heger T , Mancini R , Herzog F , Kartenbeck J , Hayer A , Helenius A . Role of endosomes in simian virus 40 entry and infection. J Virol 2011;85:4198–4211. 21345959 10.1128/JVI.02179-10 PMC3126231

[49] Szklarczyk D , Franceschini A , Kuhn M , Simonovic M , Roth A , Minguez P , Doerks T , Stark M , Muller J , Bork P , Jensen LJ , von Mering C . The STRING database in 2011: functional interaction networks of proteins, globally integrated and scored. Nucleic Acids Res 2011;39:D561–D568. 21045058 10.1093/nar/gkq973 PMC3013807

[50] Huang da W , Sherman BT , Lempicki RA . Systematic and integrative analysis of large gene lists using DAVID bioinformatics resources. Nat Protoc 2009;4:44–57. 19131956 10.1038/nprot.2008.211

[51] Dennis G Jr , Sherman BT , Hosack DA , Yang J , Baseler MW , Lane HC , Lempicki RA . DAVID: database for annotation, visualization, and integrated discovery. Genome Biol 2003;4:3. PMC193660 12734009

[52] Yoshida S , Hoppe AD , Araki N , Swanson JA . Sequential signaling in plasma‐membrane domains during macropinosome formation in macrophages. J Cell Sci 2009;122:3250–3261. 19690049 10.1242/jcs.053207 PMC2736863

[53] Zoncu R , Perera RM , Balkin DM , Pirruccello M , Toomre D , De Camilli P . A phosphoinositide switch controls the maturation and signaling properties of APPL endosomes. Cell 2009;136:1110–1121. 19303853 10.1016/j.cell.2009.01.032 PMC2705806

[54] de Lartigue J , Polson H , Feldman M , Shokat K , Tooze SA , Urbe S , Clague MJ . PIKfyve regulation of endosome‐linked pathways. Traffic 2009;10:883–893. 19582903 10.1111/j.1600-0854.2009.00915.x PMC2723830

[55] Schmidt FI , Bleck CK , Reh L , Novy K , Wollscheid B , Helenius A , Stahlberg H , Mercer J . Vaccinia virus entry is followed by core activation and proteasome‐mediated release of the immunomodulatory effector VH1 from lateral bodies. Cell Rep 2013;4:464–476. 23891003 10.1016/j.celrep.2013.06.028

[56] Smith GL , Vanderplasschen A , Law M . The formation and function of extracellular enveloped vaccinia virus. J Gen Virol 2002;83:2915–2931. 12466468 10.1099/0022-1317-83-12-2915

[57] Perini ED , Schaefer R , Stoter M , Kalaidzidis Y , Zerial M . Mammalian CORVET is required for fusion and conversion of distinct early endosome subpopulations. Traffic 2014;15:1366–1389. 25266290 10.1111/tra.12232

[58] Bogdanovic A , Bennett N , Kieffer S , Louwagie M , Morio T , Garin J , Satre M , Bruckert F . Syntaxin 7, syntaxin 8, Vti1 and VAMP7 (vesicle‐associated membrane protein 7) form an active SNARE complex for early macropinocytic compartment fusion in Dictyostelium discoideum . Biochem J 2002;368:29–39. 12175335 10.1042/BJ20020845 PMC1222979

[59] Xu Y , Hortsman H , Seet L , Wong SH , Hong W . SNX3 regulates endosomal function through its PX‐domain‐mediated interaction with PtdIns(3)P. Nat Cell Biol 2001;3:658–666. 11433298 10.1038/35083051

[60] Humphries WHt , Szymanski CJ , Payne CK . Endo‐lysosomal vesicles positive for Rab7 and LAMP1 are terminal vesicles for the transport of dextran. PLoS One 2011;6:e26626. 22039519 10.1371/journal.pone.0026626 PMC3200357

[61] Schmidt FI , Kuhn P , Robinson T , Mercer J , Dittrich PS . Single‐virus fusion experiments reveal proton influx into vaccinia virions and hemifusion lag times. Biophys J 2013;105:420–431. 23870263 10.1016/j.bpj.2013.06.016 PMC3714929

[62] Laliberte JP , Weisberg AS , Moss B . The membrane fusion step of vaccinia virus entry is cooperatively mediated by multiple viral proteins and host cell components. PLoS Pathog 2011;7:e1002446. 22194690 10.1371/journal.ppat.1002446 PMC3240603

[63] Pankiv S , Alemu EA , Brech A , Bruun JA , Lamark T , Overvatn A , Bjorkoy G , Johansen T . FYCO1 is a Rab7 effector that binds to LC3 and PI3P to mediate microtubule plus end‐directed vesicle transport. J Cell Biol 2010;188:253–269. 20100911 10.1083/jcb.200907015 PMC2812517

[64] Wang T , Ming Z , Xiaochun W , Hong W . Rab7: role of its protein interaction cascades in endo‐lysosomal traffic. Cell Signal 2011;23:516–521. 20851765 10.1016/j.cellsig.2010.09.012

[65] Wang T , Hong W . Interorganellar regulation of lysosome positioning by the Golgi apparatus through Rab34 interaction with Rab‐interacting lysosomal protein. Mol Biol Cell 2002;13:4317–4332. 12475955 10.1091/mbc.E02-05-0280 PMC138636

[66] Kruth HS , Jones NL , Huang W , Zhao B , Ishii I , Chang J , Combs CA , Malide D , Zhang WY . Macropinocytosis is the endocytic pathway that mediates macrophage foam cell formation with native low density lipoprotein. J Biol Chem 2005;280:2352–2360. 15533943 10.1074/jbc.M407167200

[67] Levin R , Grinstein S , Schlam D . Phosphoinositides in phagocytosis and macropinocytosis. Biochim Biophys Acta 2015;1851:805–823. 25238964 10.1016/j.bbalip.2014.09.005

[68] Soares JA , Leite FG , Andrade LG , Torres AA , De Sousa LP , Barcelos LS , Teixeira MM , Ferreira PC , Kroon EG , Souto‐Padron T , Bonjardim CA . Activation of the PI3K/Akt pathway early during vaccinia and cowpox virus infections is required for both host survival and viral replication. J Virol 2009;83:6883–6899. 19386722 10.1128/JVI.00245-09 PMC2698574

[69] McNulty S , Bornmann W , Schriewer J , Werner C , Smith SK , Olson VA , Damon IK , Buller RM , Heuser J , Kalman D . Multiple phosphatidylinositol 3‐kinases regulate vaccinia virus morphogenesis. PLoS One 2010;5:e10884. 20526370 10.1371/journal.pone.0010884 PMC2878334

[70] Izmailyan R , Hsao JC , Chung CS , Chen CH , Hsu PW , Liao CL , Chang W . Integrin beta1 mediates vaccinia virus entry through activation of PI3K/Akt signaling. J Virol 2012;86:6677–6687. 22496232 10.1128/JVI.06860-11 PMC3393588

[71] Hiley CT , Chard LS , Gangeswaran R , Tysome JR , Briat A , Lemoine NR , Wang Y . Vascular endothelial growth factor A promotes vaccinia virus entry into host cells via activation of the Akt pathway. J Virol 2013;87:2781–2790. 23269798 10.1128/JVI.00854-12 PMC3571386

[72] Er EE , Mendoza MC , Mackey AM , Rameh LE , Blenis J . AKT facilitates EGFR trafficking and degradation by phosphorylating and activating PIKfyve. Sci Signal 2013;6:ra45. 23757022 10.1126/scisignal.2004015 PMC4041878

[73] Moss B . Poxvirus cell entry: how many proteins does it take? Viruses 2012;4:688–707. 22754644 10.3390/v4050688 PMC3386626

[74] Fairn GD , Grinstein S . How nascent phagosomes mature to become phagolysosomes. Trends Immunol 2012;33:397–405. 22560866 10.1016/j.it.2012.03.003

[75] Hyttinen JM , Niittykoski M , Salminen A , Kaarniranta K . Maturation of autophagosomes and endosomes: a key role for Rab7. Biochim Biophys Acta 2013;1833:503–510. 23220125 10.1016/j.bbamcr.2012.11.018

[76] Pelkmans L , Burli T , Zerial M , Helenius A . Caveolin‐stabilized membrane domains as multifunctional transport and sorting devices in endocytic membrane traffic. Cell 2004;118:767–780. 15369675 10.1016/j.cell.2004.09.003

