# Supplementary material for: Vaccinia Virus Infection Requires Maturation of Macropinosomes
Source: Traffic. 2015 May 6;16(8):814–31. doi: 10.1111/tra.12290 (PMC4973667; doi:10.1111/tra.12290)
Supplement: Supplementary file 1 — Figure S1: Automated analysis of VACV internalization and endosome colocalization. A) Detection of endocytic vesicles (endosomes) and viral particles using the imaris software. Digital images of endocytic vesicles, total virus particles (VACV tot) and external virus particles (VACV ext) generated using the imaris spot detection are displayed. B) Automatic detection of internalized virions (VACV int) with imaris. For this, internalized mCherry‐positive virus particles were defined as those that have no associated VACV ext fluorescence. C) The imaris ‘spot colocalization’ tool was used to automatically detect the colocalization between internalized virions (VACV int) and endocytic vesicles (endosomes). A virion and an endocytic vesicle were considered to colocalize when their distance (from the center or each spot) was ≤200 nm. White arrowheads indicate internalized virus particles that colocalize with endosomal vesicles. Non‐internalized virions (VACV ext) were used as negative controls (light‐blue arrowhead). [file TRA-16-814-s001.doc]

**
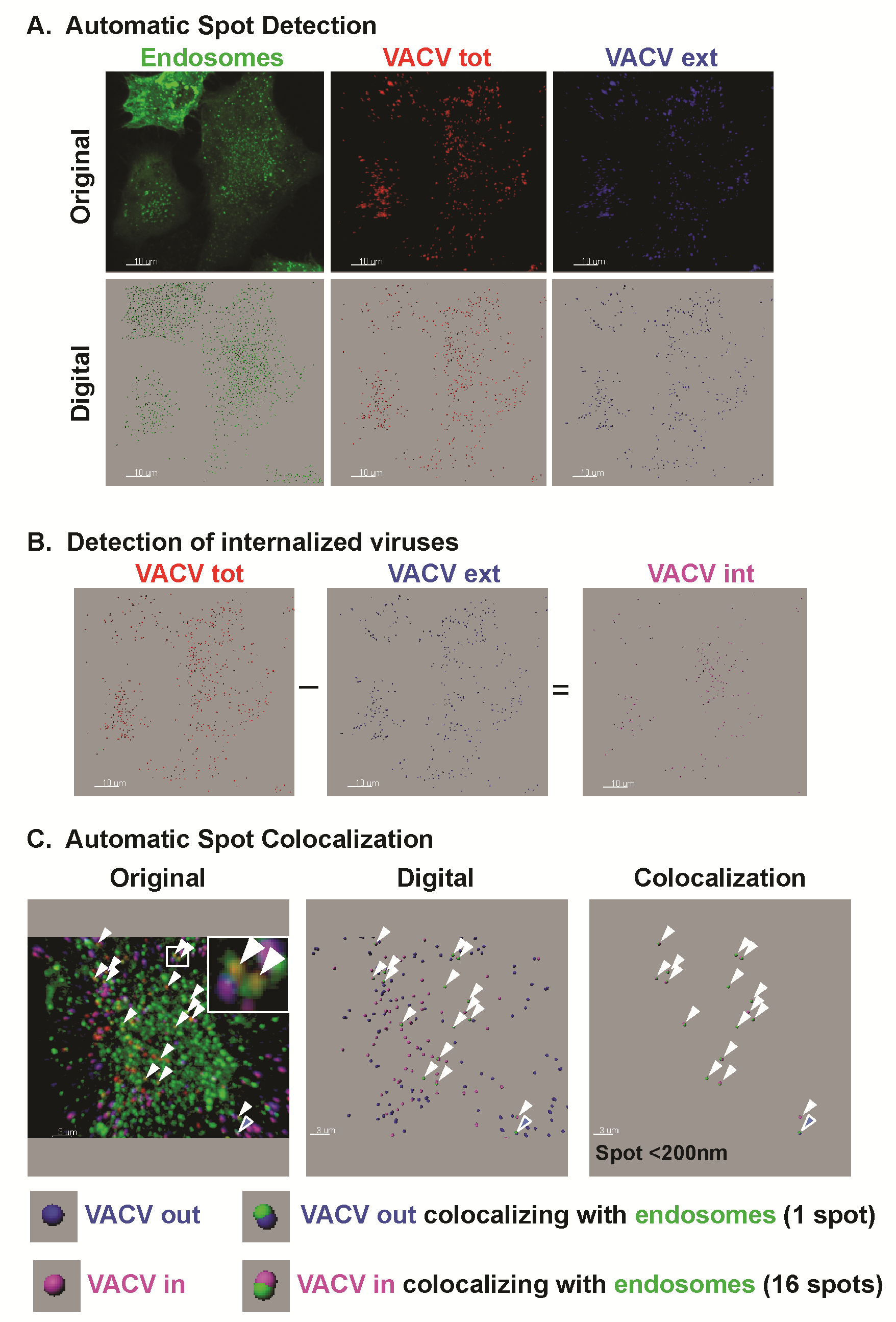
**

**Figure S1: Automated analysis of VACV virus internalization and endosome colocalization.** A) Detection of endocytic vesicles (endosomes) and viral particles and using the Imaris software. Digital images of endocytic vesicles, total virus particles (VACV tot), and external virus particles (VACV ext) generated using the Imaris Spot Detection are displayed. B) Automatic detection of internalized virions (VACV int) with Imaris. For this, internalized mCherry-positive virus particles were defined as those that have no associated VACV ext fluorescence. C) The Imaris 'spot colocalization' tool was used to automatically detect the colocalization between internalized virions (VACV int) and endocytic vesicles (endosomes). A virion and an endocytic vesicle were considered to colocalize when their distance (from the center or each spot) was ≤ 200 nm. White arrowheads indicate internalized virus particles that colocalize with endosomal vesicles. Non-internalized virions (VACV ext) were used as negative controls (light-blue arrowhead).
