# Supplementary material for: Vaccinia Virus Infection Requires Maturation of Macropinosomes
Source: Traffic. 2015 May 6;16(8):814–31. doi: 10.1111/tra.12290 (PMC4973667; doi:10.1111/tra.12290)
Supplement: Supplementary file 2 — Figure S2: Colocalization of VACV with endogenous Rab5, Rab7 and LAMP1. A–C) HeLa cells were bound with VACV WR mCherry‐A4 MVs at an MOI of 2 at 4°C. Cells were washed and shifted to 37°C for the indicated time points. Non‐permeabilized cells were then subjected to immunostaining with α‐L1R to distinguish external (blue) versus internalized (red) virions. To visualize endogenous Rab5 (A), Rab7 (B) or LAMP1 (C), cells were permeabilized and immunostained using antibodies directed against these various markers. Insets display colocalization events in the xy, yz and xz planes. White arrows represent colocalization events. D) The percent colocalization between internalized virions and the various endocytic markers was determine using imaris automated colocalization analysis as described in Figure S1. At least 30 total cells from three independent experiments were analyzed for each marker. Results displayed as the average ± SD. [file TRA-16-814-s002.doc]

**
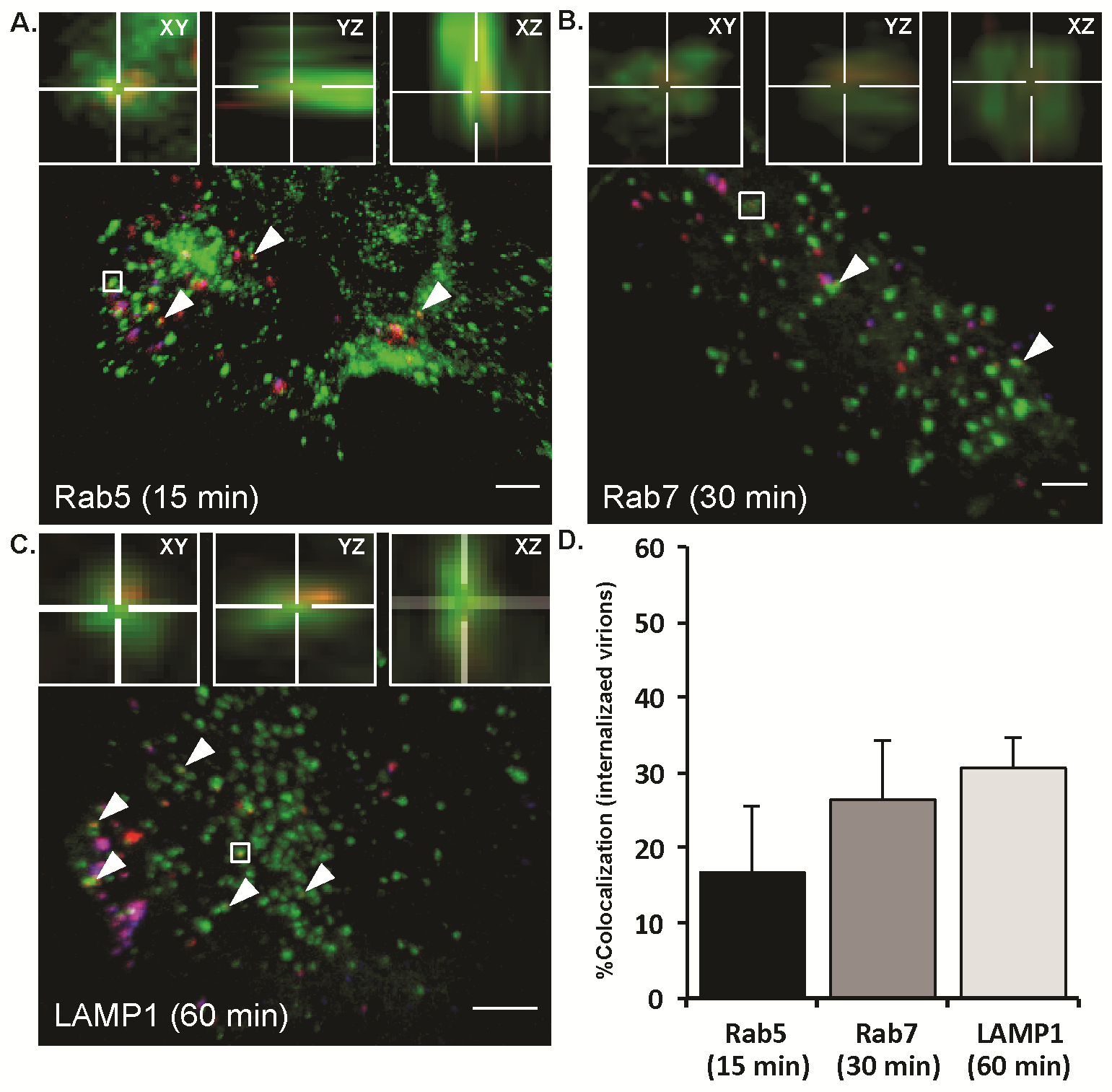
**

**Figure S2: Colocalization of VACV with endogenous Rab5, Rab7, and LAMP1.** A-C) HeLa cells were bound with VACV WR mCherry-A4 MVs at an MOI of 2 at 4 ^o^C. Cells were washed and shifted to 37 ^o^C for the indicated time points. Non-permeabilized cells were then subjected to immunostaining with α-L1R to distinguish external (blue) vs. internalized (red) virions. To visualize endogenous Rab5 (A), Rab7 (B), or LAMP1 (C), cells were permeabilized and immunostained using antibodies directed against these various markers. Insets display colocalization events in the xy, yz, and xz planes. White arrows represent colocalization events. D) The percent colocalization between internalized virions and the various endocytic markers was determine using Imaris Automated coloclaization analysis as described in Figure S1. At least 30 total cells from 3 independent experiments were analyzed for each marker. Results displayed as the average ± STDV.
