# Supplementary material for: Vaccinia Virus Infection Requires Maturation of Macropinosomes
Source: Traffic. 2015 May 6;16(8):814–31. doi: 10.1111/tra.12290 (PMC4973667; doi:10.1111/tra.12290)
Supplement: Supplementary file 3 — Figure S3: RNAi screen workflow, image analysis and cell number correction. A) The ‘usual suspects’ siRNA library consists of two 384‐well plates. Three copies of the library (six plates in total) were used in each experiment. The siRNAs were introduced into HeLa ATCC cells by reverse transfection. At 72 h post‐transfection, cells were infected with WR E EGFP MVs. At 6 h p.i., cells were fixed, nuclei stained with DAPI and the EGFP signal was enhanced by immunofluorescence staining using an α‐EGFP antibody. Assay plates were then imaged using an image xpress Microscreening system. The screen was repeated three independent times and the results were shown as the mean of the triplicates. B) Image analysis was performed using an in‐house matlab‐based software that allowed for automatic digital detection and scoring of nuclei and EGFP‐positive infected cells (scale bars, 50 µm). C) To correct for the effect on infection index due to deleterious effects of RNAi transfection on cell number variability, an infection index checkerboard was used for correction. Infection of a gradient of cells treated with control siRNA (AllStarNegative) was used to determine the correlation between the number of cells and the corresponding infection index. This was then used to create a normalization curve that was applied to the screening data to eliminate any cell number bias on infection. Any siRNA target wells displaying <200 cells were discarded from the analysis. [file TRA-16-814-s003.doc]

**
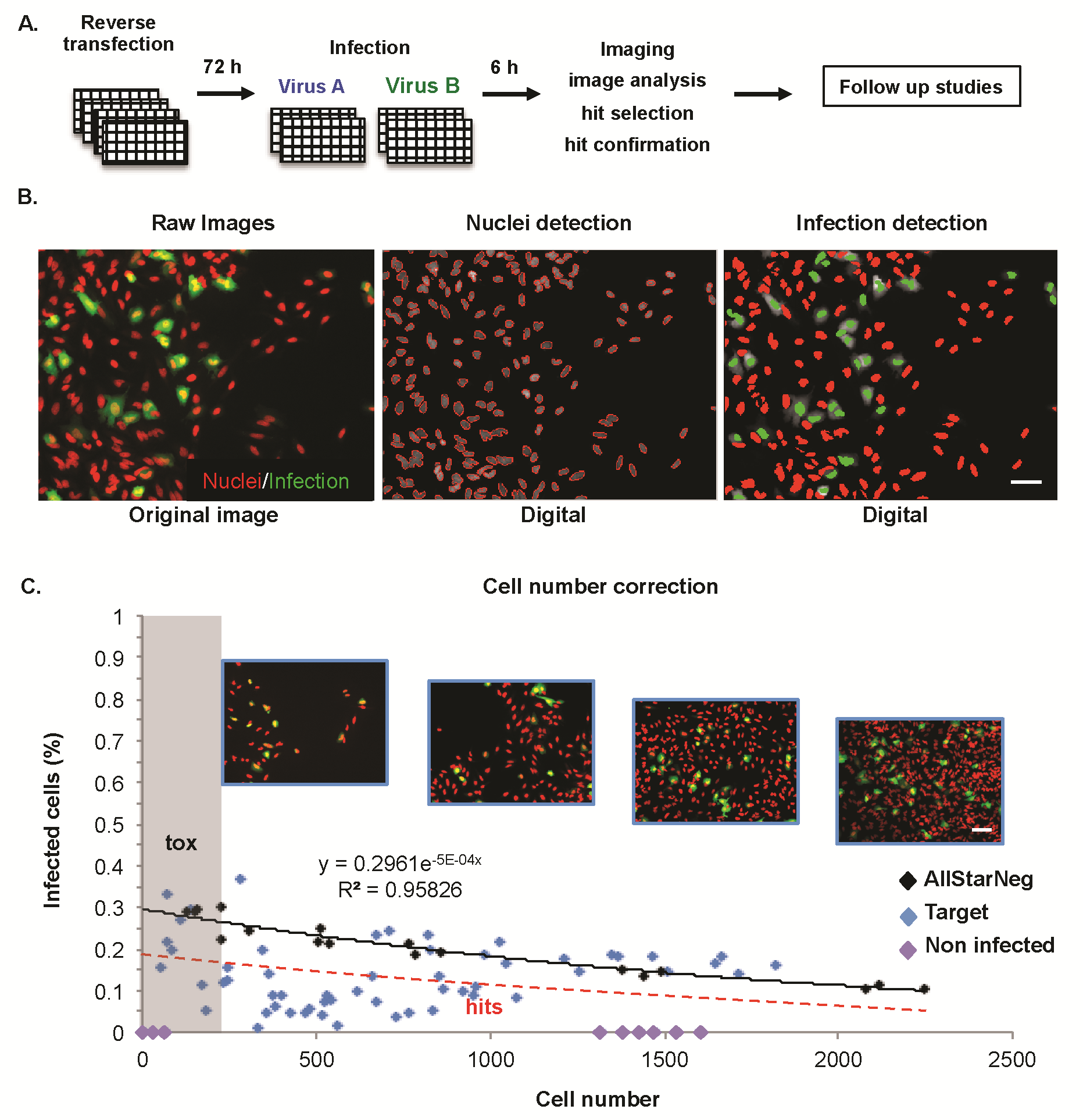
**

**Figure S3: RNAi screen workflow, image analysis, and cell number correction**. A) The "Usual Suspects" siRNA library consists of two 384-well plates. Three copies of the library (6 plates in total) were used in each experiment. The siRNAs were introduced into Hela ATCC cells by reverse-transfection. At 72 h post transfection cells were infected with WR E EGFP MVs. At 6 h p.i. cells were fixed, nuclei stained with DAPI, and the EGFP signal enhanced by immunofluorescence staining using a α−EGFP antibody. Assay plates were then imaged using a ImageXpress Micro screening system. The screen was repeated three independent times and the results shown as the mean of the triplicates. B) Image analysis was performed using an in-house Matlab-based software that allowed for automatic digital detection and scoring of nuclei and EGFP positive infected cells. (Scale bars,  50μm). C) To correct for the impact on infection index due to deleterious effects of RNAi transfection on cell number variability, an infection index checkerboard was used for correction. Infection of a gradient of cells treated with control siRNA (AllStarNegative) was used to determine to the correlation between the number of cells and the corresponding infection index. This was then used to create a normalization curve that was applied to the screening data to eliminate any cell number bias on infection. Any siRNA target wells displaying less than 200 cells were discarded from the analysis.
