# Supplementary material for: Vaccinia Virus Infection Requires Maturation of Macropinosomes
Source: Traffic. 2015 May 6;16(8):814–31. doi: 10.1111/tra.12290 (PMC4973667; doi:10.1111/tra.12290)
Supplement: Supplementary file 4 — Figure S4: Colocalization of VACV MVs with SNX3. Cells transfected with EGFP‐SNX3 were infected with WR mCherry‐A4 MVs at an MOI of 2. At the indicated time points, cells were fixed and non‐permeabilized cells were subjected to immunostaining with α‐L1R to distinguish bound virions (purple). White arrows represent colocalization events and representative images of the peak time points of colocalization are displayed. Insets display individual colocalization examples from boxed regions in xy, yz and xz planes (imaris). Bars, 5 µm. [file TRA-16-814-s004.doc]

**
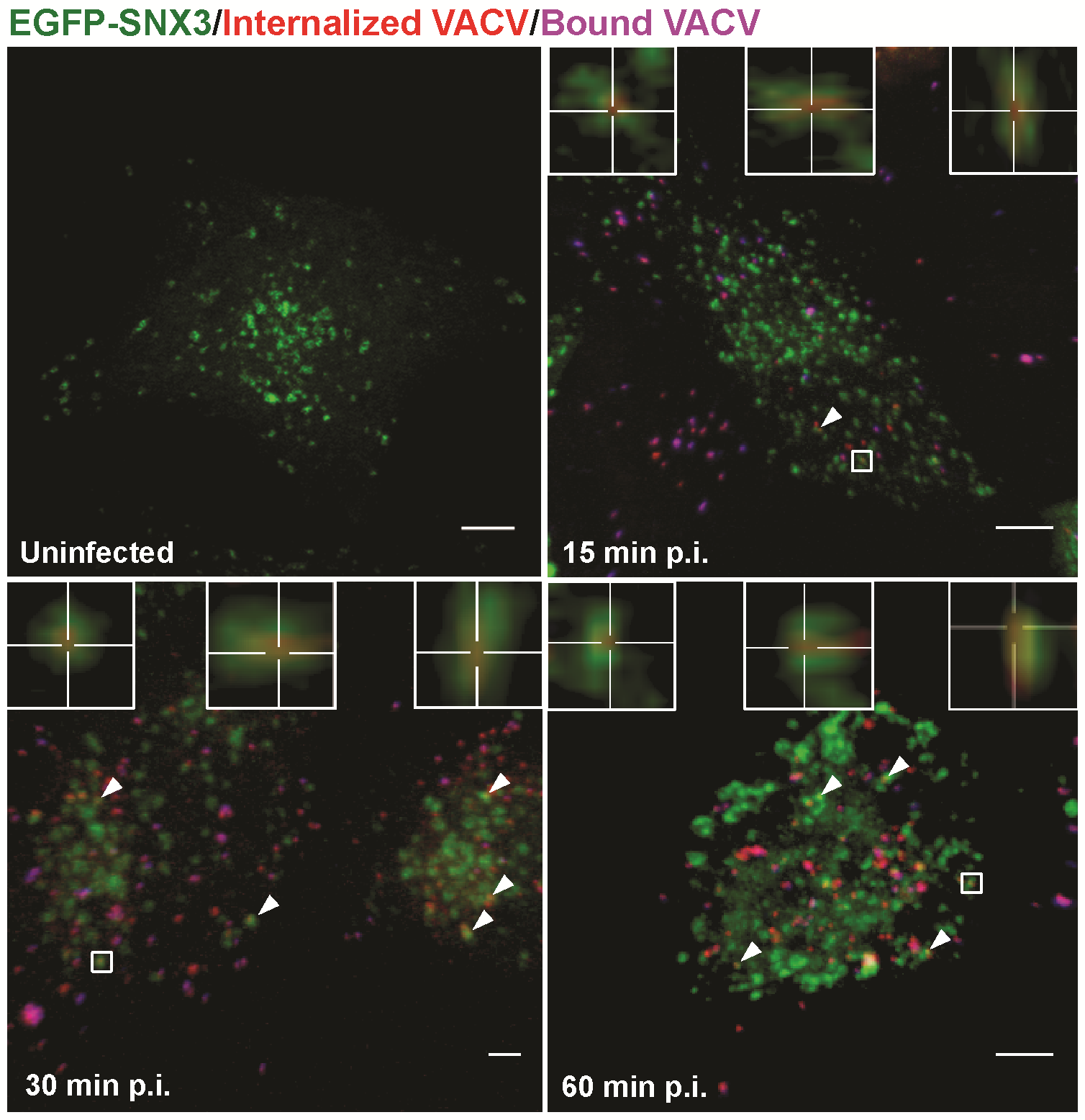
**

**Figure S4: Colocalization of VACV MVs with SNX3.** Cells transfected with EGFP-SNX3 were infected with WR mCherrry-A4 MVs at an MOI of 2. At the indicated times cells were fixed and non-permeabilized cells subjected to immunostaining with α-L1R to distinguish bound virions (purple). White arrows represent colocalization events and representative images of the peak time points of colocalization are displayed. Insets display individual co-localization examples from boxed regions in xy, yz, and xz planes (Imaris). Bars; 5µm.
