# Supplementary material for: Vaccinia Virus Infection Requires Maturation of Macropinosomes
Source: Traffic. 2015 May 6;16(8):814–31. doi: 10.1111/tra.12290 (PMC4973667; doi:10.1111/tra.12290)
Supplement: Supplementary file 5 — Figure S5: VACV MV infection relies on Rab34 function. HeLa cells were transfected with WT, C/A or D/N versions of EGFP‐Rab34. At 18 h p.i., cells were infected with WR E/L mRFP MVs. Cells were harvested for flow cytometry, and 10 000 transfected cells were scored for infection. Results are displayed as the percent infection relative to infection of WT Rab34 overexpressing cells and represent the means of three independent experiments ± SD. [file TRA-16-814-s005.doc]

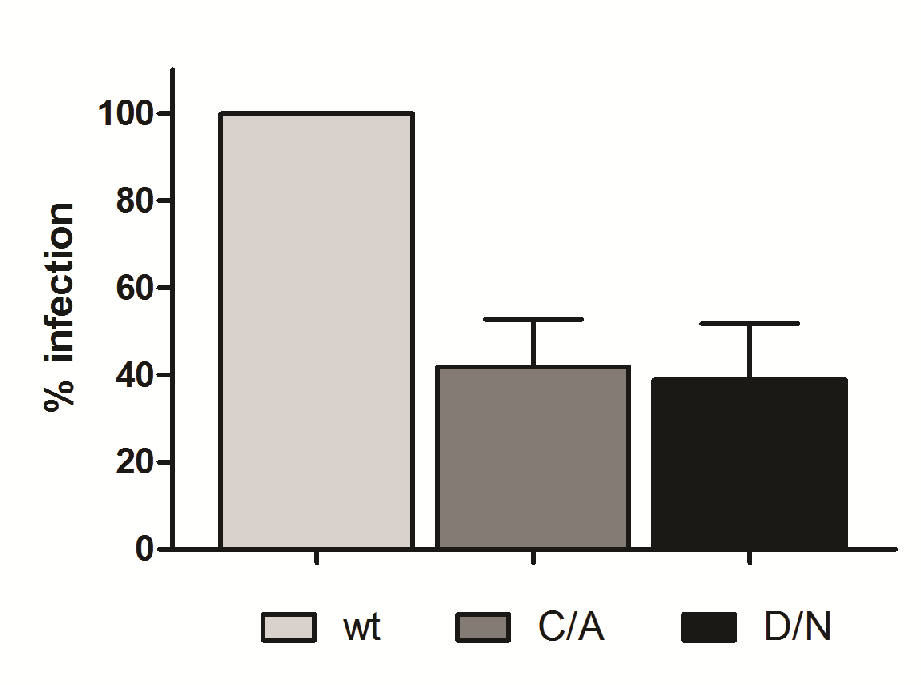


**Figure S5: VACV MV infection relies on Rab34 function.** HeLa cells were transfected with WT, C/A, or D/N versions of EGFP-Rab34. At 18 h p.i. cells were infected with WR E/L mRFP MVs. Cells were harvested for flow cytometry, ten thousand transfected cells were scored for infection. Results are displayed as the percent infection relative to infection of WT Rab34 overexpressing cells and represent the means of 3 independent experiments ±STDV.
