# Supplementary material for: Vaccinia Virus Infection Requires Maturation of Macropinosomes
Source: Traffic. 2015 May 6;16(8):814–31. doi: 10.1111/tra.12290 (PMC4973667; doi:10.1111/tra.12290)
Supplement: Supplementary file 6 — Figure S6: VACV MV infection does not require MT dynamics. HeLa cells were pre‐treated with the indicated compounds at 10 µm for 1 h prior to infection. Cells were then infected with WR E EGFP L mCherry virus (MOI = 2). At 12 h p.i., cells were harvested and analyzed by flow cytometry for both EGFP (black bars; early gene expression) and mCherry (gray bars; late gene expression). The average of two independent experiments is displayed as percent infection relative to control infections set at 100%. [file TRA-16-814-s006.doc]

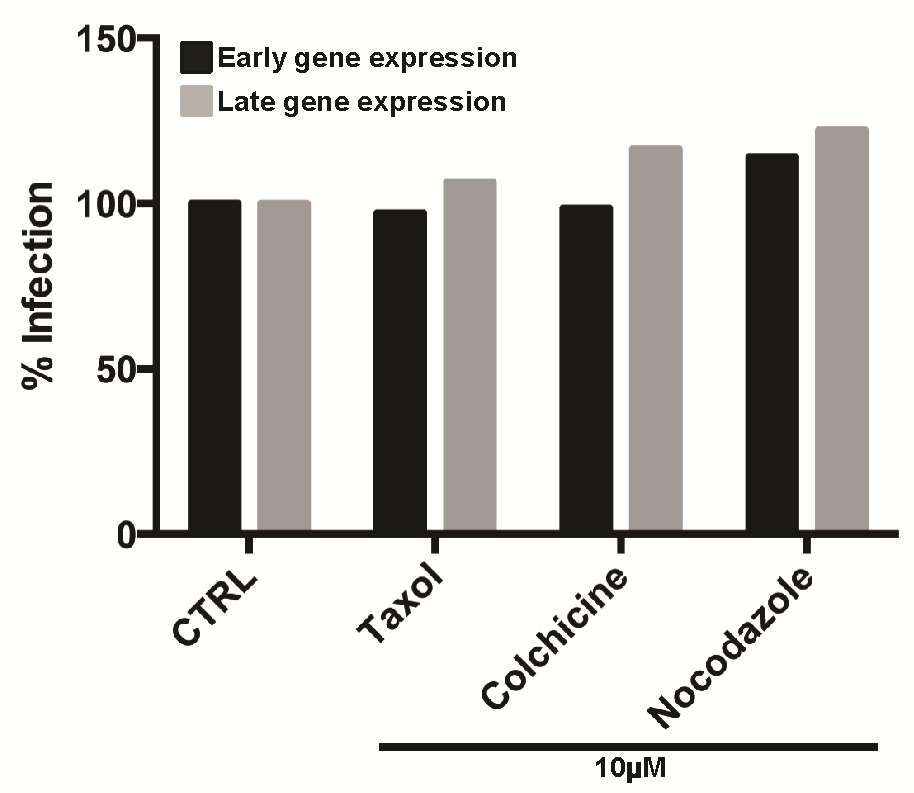


**Figure S6: VACV MV infection does not require MT dynamics.** Hela cells were pre-treated with the indicated compounds at 10µM for 1 hour prior to infection. Cells were then infected with WR E EGFP L mCherry virus (MOI 2). At 12 h post infection cells were harvested and analyzed by flow cytometry for both EGFP (black bars; early gene expression) and mCherry (grey bars; late gene expression). The average of two independent experiments is displayed as % infection relative to control infections set at 100%.
